# Myostatin inhibition prevents skeletal muscle pathophysiology in Huntington’s disease mice

**DOI:** 10.1038/s41598-017-14290-3

**Published:** 2017-10-27

**Authors:** Marie K. Bondulich, Nelly Jolinon, Georgina F. Osborne, Edward J. Smith, Ivan Rattray, Andreas Neueder, Kirupa Sathasivam, Mhoriam Ahmed, Nadira Ali, Agnesska C. Benjamin, Xiaoli Chang, James R. T. Dick, Matthew Ellis, Sophie A. Franklin, Daniel Goodwin, Linda Inuabasi, Hayley Lazell, Adam Lehar, Angela Richard-Londt, Jim Rosinski, Donna L. Smith, Tobias Wood, Sarah J. Tabrizi, Sebastian Brandner, Linda Greensmith, David Howland, Ignacio Munoz-Sanjuan, Se-Jin Lee, Gillian P. Bates

**Affiliations:** 1 Sobell Department of Motor Neuroscience and Movement Disorders, University College London Institute of Neurology, London, WC1N 3BG UK; 2 Department Medical and Molecular Genetics, King’s College London, London, SE1 9RT UK; 3 Huntington’s Disease Centre, UCL Institute of Neurology, London, WC1N 3BG UK; 4 MRC Centre for Neuromuscular Diseases, UCL Institute of Neurology, London, WC1N 3BG UK; 5 Department Molecular Biology and Genetics, The Johns Hopkins University School of Medicine, Baltimore, MD 21205 USA; 6 Division of Neuropathology, UCL Institute of Neurology, London, WC1N 3BG UK; 7 Department of Neurodegenerative disease, UCL Institute of Neurology, London, WC1N 3BG UK; 8 CHDI Management/CHDI Foundation Inc, New York, NY 10001 USA; 9 Department of Neuroimaging, King’s College London, Institute of Psychiatry, London, SE5 8AF UK

## Abstract

Huntington’s disease (HD) is an inherited neurodegenerative disorder of which skeletal muscle atrophy is a common feature, and multiple lines of evidence support a muscle-based pathophysiology in HD mouse models. Inhibition of myostatin signaling increases muscle mass, and therapeutic approaches based on this are in clinical development. We have used a soluble ActRIIB decoy receptor (ACVR2B/Fc) to test the effects of myostatin/activin A inhibition in the R6/2 mouse model of HD. Weekly administration from 5 to 11 weeks of age prevented body weight loss, skeletal muscle atrophy, muscle weakness, contractile abnormalities, the loss of functional motor units in EDL muscles and delayed end-stage disease. Inhibition of myostatin/activin A signaling activated transcriptional profiles to increase muscle mass in wild type and R6/2 mice but did little to modulate the extensive Huntington’s disease-associated transcriptional dysregulation, consistent with treatment having little impact on HTT aggregation levels. Modalities that inhibit myostatin signaling are currently in clinical trials for a variety of indications, the outcomes of which will present the opportunity to assess the potential benefits of targeting this pathway in HD patients.

## Introduction

Huntington’s disease (HD) is an inherited progressive neurodegenerative disorder for which the age of onset is generally in midlife. It is caused by a CAG repeat expansion in the first exon of the huntingtin (*HTT*) gene that results in an abnormally long polyglutamine tract in the huntingtin protein (HTT)^1^. Symptoms include problems with motor coordination, neuropsychiatric symptoms and cognitive decline; and there are no disease-modifying treatments^2^. The mutant form of the HTT protein aggregates into oligomeric and fibrillary structures which are deposited in the brains of HD patients^3,4^ and neurodegeneration occurs in the striatum, cortex, hypothalamus and other brain regions^5^. There is increasing evidence to indicate that Huntington’s disease is also associated with pathological processes that occur in peripheral tissues, including skeletal muscle^6^. Treatments targeted to tissues and organs outside the CNS have the potential to substantially improve the quality of life of HD patients, either in the absence of disease modifying treatments or combined with CNS-targeted therapies^7^.

Mouse models of HD include mice that are transgenic for the 5′ region of the human *HTT* gene (e.g. R6/2), those transgenic for the entire human *HTT* gene (e.g. BACHD and YAC128) and a range of knock-in models (e.g. *Hdh*Q150 and zQ175)^8^. In recent years, we have performed detailed comparative analyses of the R6/2 mouse model^9^ and *Hdh*Q150 knock-in mice^10^ and found that at late stage disease (14 weeks for R6/2 and 22 months for homozygous *Hdh*Q150) the phenotypes of these two models are extremely similar^11–16^, the main difference between the lines being the age at phenotype onset and the rate of disease progression. The R6/2 mouse is transgenic for a genomic DNA fragment encoding exon 1 of human *HTT* and is a model of the incomplete splicing event that occurs in all HD knock-in mouse models, YAC128 and BACHD mice^17^, and in HD patient tissues^18^, resulting in an exon 1 – intron 1 mRNA that in all cases produces the highly pathogenic exon 1 HTT protein. Both the R6/2 and *Hdh*Q150 models develop a progressive failure to gain body weight, followed by weight loss^11^ and a highly comparable skeletal muscle atrophy and underlying muscle pathophysiology^19^. The skeletal muscle phenotype manifests as a uniform muscle fibre atrophy^19–21^ and functional deficits^19,22^, associated with the formation of nuclear inclusions^13,20^, transcriptional dysregulation^19,23–25^, energetic disturbances^19,25^, ion channel perturbations^26^, alterations in protein synthesis and degradation pathways^27^ and an apparent dissociation of trophic signaling between motor neurons and skeletal muscle^21^. The investigation of the mechanisms underlying muscle pathology in HD mouse models is likely to lead to insights into the muscle atrophy and loss of muscle strength that has been reported in HD patients (for review, see^28^).

Myostatin is a member of the transforming growth factor β super family of secreted growth factors that is synthesized as a full length precursor and becomes cleaved into an amino-terminal propeptide and carboxy terminal mature region^29^. It has been shown to be a muscle specific regulator of muscle size, and the knock-out of myostatin results in muscle hypertrophy and a reduction in adipose tissue mass^30,31^. Binding of myostatin and activin A to the activin A receptor type IIB (ActRIIB) on muscle cell membranes, activates SMAD 2/3-mediated transcription, which stimulates FOXO-dependent transcription increasing muscle protein degradation. SMAD activation also inhibits muscle protein synthesis by suppressing AKT signaling (for review see^32^). A soluble form of the activin type IIB receptor (ACVR2B/Fc) has been developed to compete with the natural receptor for circulating agonists and this caused a dramatic increase in muscle mass when injected into wild type (WT) mice^33^. Therefore, agents that block the myostatin signaling pathway have the potential to treat human disease associated with cachexia e.g. cancer, chronic kidney disease and chronic heart failure, as well as neuromuscular diseases^32^.

We have used the ACVR2B/Fc soluble receptor to test whether the inhibition of myostatin/activin A signaling might prevent or decrease skeletal muscle atrophy and restore muscle function in mouse models of HD. Administration of ACVR2B/Fc to R6/2 mice from 5 weeks of age, completely prevented deficits in body weight gain, muscle atrophy, grip strength, muscle function and motor unit loss and also delayed end-stage disease. A range of therapeutic agents that inhibit myostatin signaling are currently being tested in clinical trials for a number of indications^34^, the outcomes of which will provide the opportunity to design clinical trials to treat muscle atrophy in people affected with HD.

## Results

### Treatment with ACVR2B/Fc prevents body weight loss, grip strength deficits and muscle atrophy in R6/2 mice

**Figure 1:**
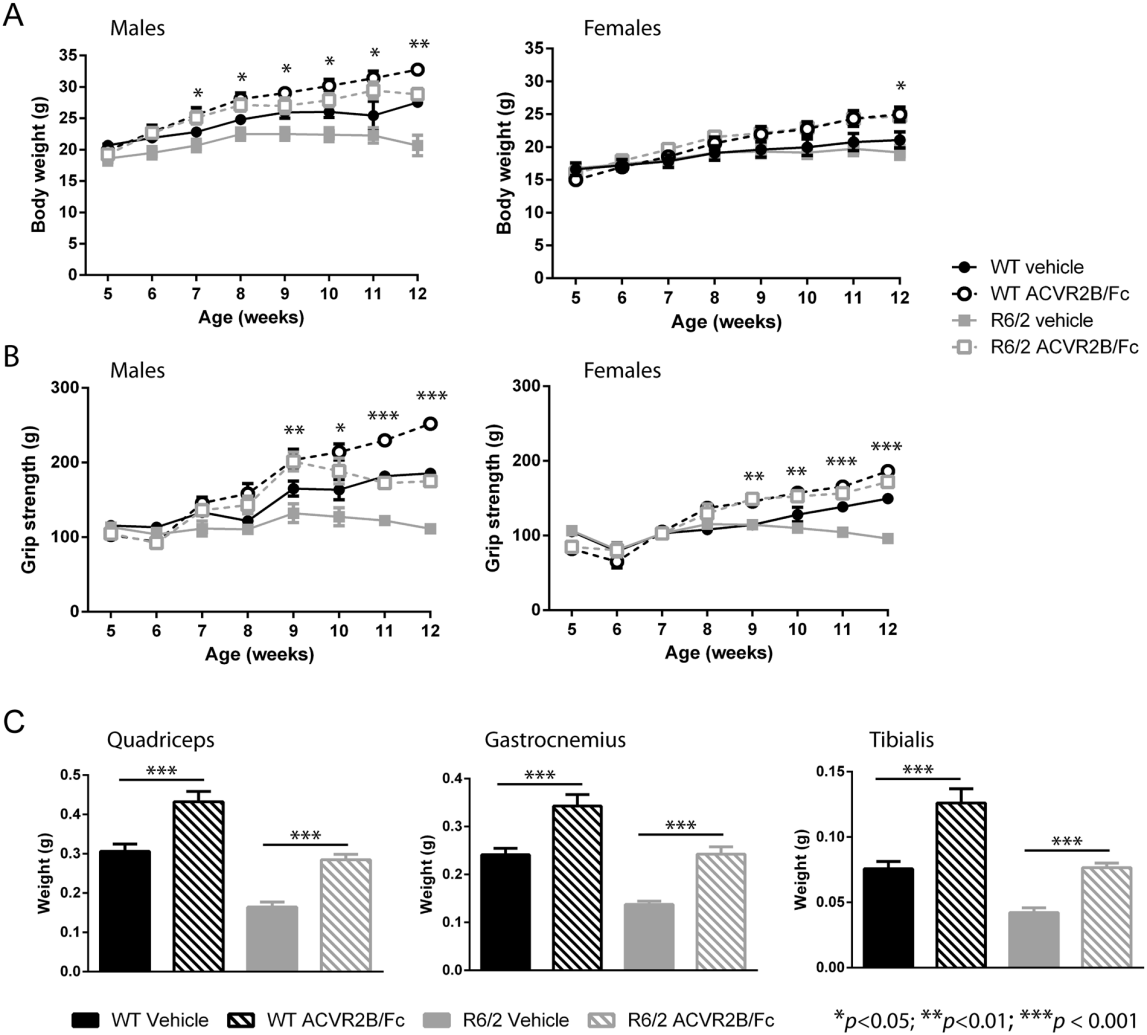
Treatment with ACVR2B/Fc restores deficits in body weight, grip strength and muscle mass in R6/2 mice. (**A**) ACVR2B/Fc treatment resulted in a progressive weight gain in both WT and R6/2 mice and prevented body weight loss in R6/2 mice. (**B**) ACVR2B/Fc treatment resulted in a progressive increase in fore-limb grip strength in both WT and R6/2 mice and prevented grip strength deficits in R6/2 mice. (**C**) Treatment with ACVR2B/Fc increased muscle mass in both WT and R6/2 mice with the consequence that the R6/2 muscle mass at 12 weeks of age (genders combined) is equivalent to that of wild type mice for quadriceps, gastrocnemius and tibialis anterior hind limb skeletal muscles. Statistical analysis was two-way ANOVA with post-hoc Bonferroni correction (see Table S8 for main effects and Table S9 for multiple comparisons). The statistical significance between values for ACVR2B/Fc treated and vehicle treated R6/2 mice is depicted: *p < 0.05; **p < 0.01; ***p < 0.001. n = 5 mice per gender per genotype. All data presented as means ± SEM.

We first set out to determine whether the inhibition of myostatin/activin A signaling might reduce the extent of muscle atrophy that occurs in the R6/2 mouse model of HD. The soluble ActRIIB receptor (ACVR2B/Fc) was administered by a subcutaneous weekly 10 mg/kg injection to both WT and R6/2 male and female mice (n = 5 per gender per genotype) from 5 to 11 weeks of age, and mice were sacrificed one week later at 12 weeks. Both male and female R6/2 mice developed a progressive failure to gain weight (Fig. 1A). Treatment with ACVR2B/Fc increased the body mass of both wild type (WT) and R6/2 mice as compared to their vehicle treated littermates and in the case of R6/2 mice, this completely prevented the decreased body weight phenotype (Fig. 1A). Similarly, fore limb grip strength was progressively impaired in both male and female R6/2 mice (Fig. 1B). Treatment with the receptor decoy resulted in an increase in fore limb grip strength in both WT and R6/2 mice as compared to vehicle treated littermates, and again completely prevented the R6/2 deficits (Fig. 1B). At sacrifice, the tibialis anterior (TA), quadriceps and gastrocnemius skeletal hind limb muscles were weighed. All three muscles showed a marked degree of atrophy in R6/2 mice (Fig. 1C). Treatment with ACVR2B/Fc increased muscle mass in both WT and R6/2 mice and, once more, in the case of R6/2, the weight of all three muscles had been restored to WT levels (Fig. 1C). In contrast, there was no effect on brain atrophy (Fig. S1A) or disease-associated changes in heart weight (Fig. S1A). Treatment with ACVR2B/Fc had been equally effective in male and female mice and, therefore, all further experiments were performed on female mice only.

To determine whether ACVR2B/Fc treatment had had an impact on body fat levels, we acquired magnetic resonance imaging (MRI) data using a 3-point Dixon technique to separate fat and water images. However, this approach did not detect a statistically significant difference between the treatment groups (Table S1).

### Treatment with ACVR2B/Fc prevents muscle fibre atrophy

**Figure 2:**
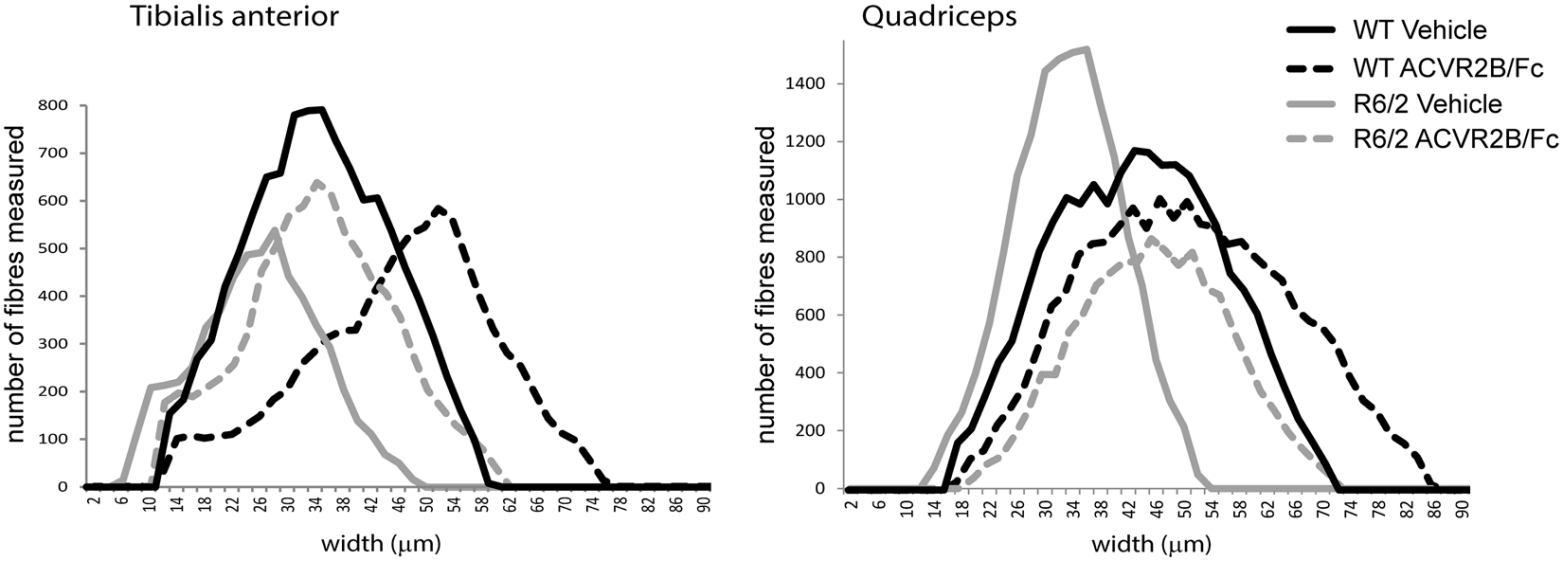
ACVR2B/Fc treatment prevents muscle fibre atrophy. (**A**) the lesser fibre diameter of myofibres in the TA and quadriceps of WT and R6/2 mice treated with ACVR2B/Fc or vehicle. Fibre counts were obtained from between 3 and 8 sections per mouse, n = 3 or 4 mice/treatment group. The WT muscle fibre diameters were greater than those for R6/2: quadriceps (F_(13,10)_ = 28.82, *p* ≤ 0.001), TA (F_(15,12)_ = 10.29, p = 0.008). ACVR2B/Fc treatment increased the fibre diameter in both cases: quadriceps (F_(13,10)_ = 92.8, *p* ≤ 0.001) and TA (F_(15,12)_ = 12.73, *p* = 0.004). ACVR2B/Fc treatment increased fibre diameter in the quadriceps (*p* < 0.001) and the TA (*p* = 0.061) of R6/2 mice. Representative traces from individual mice are depicted. Statistical analysis was two-way ANOVA with post-hoc Bonferroni correction.

The skeletal muscle atrophy that occurs in R6/2 mice has previously been studied in detail and no evidence was found of changes typically associated with muscle pathology other than a diffuse atrophy^20,21^. The adult neuromuscular innervation pattern develops normally and the incidence of abnormalities at the neuromuscular junction was very low and only occurred in animals close to end stage disease^21^. The only prominent morphological change was a severe generalized atrophy of all muscle fibres that occurred in both fibre types^20,21^. Therefore, to investigate the effect of ACVR2B/Fc treatment on muscle pathology, we measured the lesser diameters of fibres in the TA and quadriceps muscles of WT and R6/2 mice that had been treated with ACVR2B/Fc or vehicle. We found that there was a statistically significant difference in the fibre diameters between WT and R6/2 quadriceps and TA muscles (Fig. 2) and that ACVR2B/Fc treatment resulted in a statistically significant increase in the fibre diameter in both cases. Representative plots for the range of fibre diameters for the WT and R6/2 quadriceps and TA muscle treated with either vehicle or ACVR2B/Fc are illustrated in Fig. 2. ACVR2B/Fc treatment increased fibre diameter in the quadriceps (*p* < 0.001) and the TA (*p* = 0.061) of R6/2 mice.

### Treatment with ACVR2B/Fc improved muscle function in R6/2 mice

**Figure 3:**
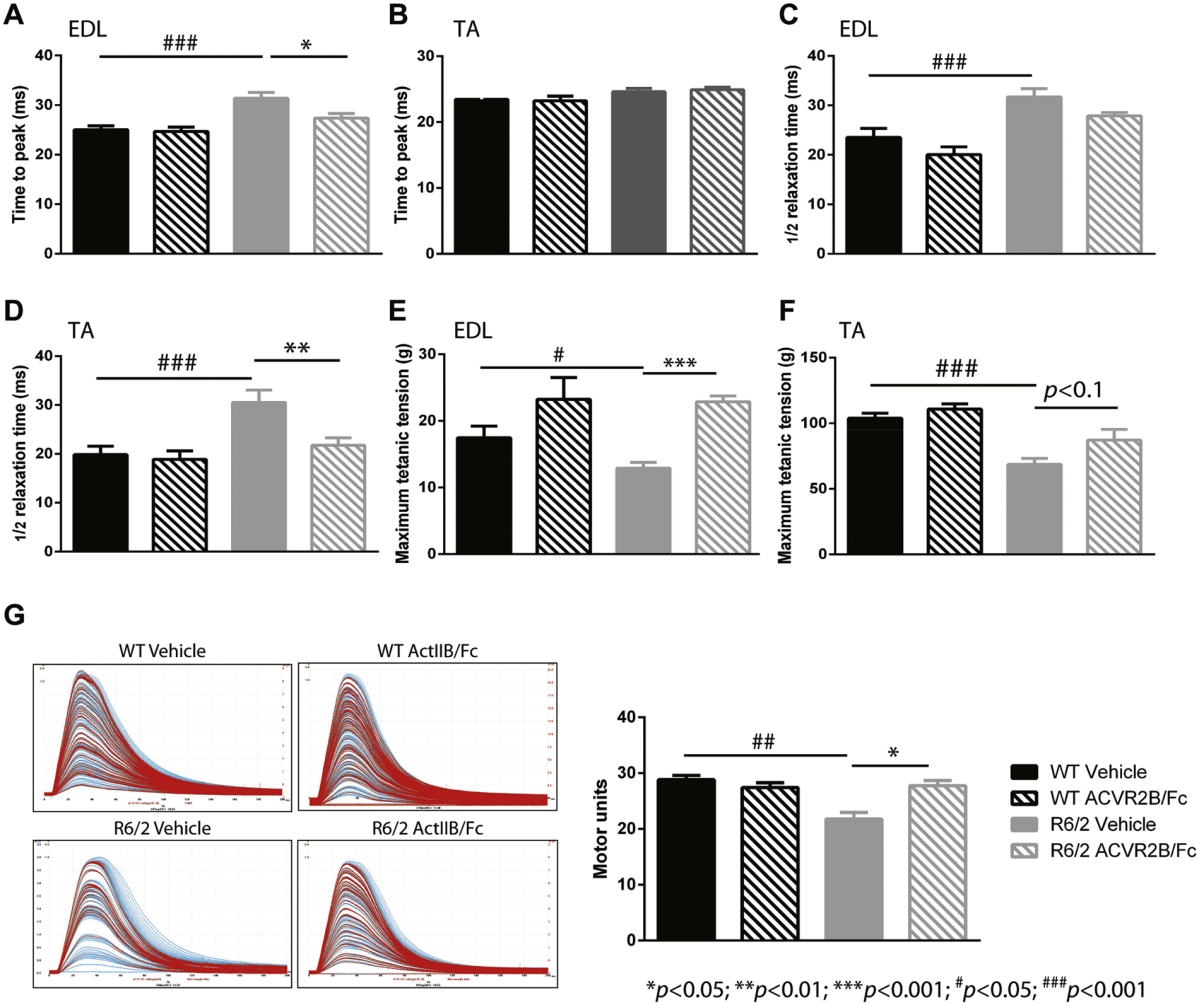
Treatment with ACVR2B/Fc improves muscle function in R6/2 mice. (**A–D**) Contractile dysfunction of the R6/2 EDL muscles was assessed by measuring twitch tension: the time to peak and half relaxation time and this was corrected by ACVR2B/Fc treatment in both EDL (**A**,**C**) and TA (**B**,**D**) muscles. (**E**,**F**) The maximum tetanic force in mice treated with ACVR2B/Fc and vehicle. The maximum EDL (**E**) and TA (**F**) forces were decreased in R6/2 mice and completely rescued in mice treated with ACVR2B/Fc. (**G**) Examples of motor unit traces and the quantification of functional motor units. The number of functional motor units was reduced in R6/2 EDL muscles and this was restored in treated mice. Statistical analysis was two-way ANOVA with post-hoc Bonferroni correction (see Table S8 for main effects and Table S9 for multiple comparisons). Statistically significant differences between vehicle treated WT and vehicle treated R6/2: ^#^ *p* < 0.05, ^###^ *p* < 0.001; statistically significant differences between ACVR2B/Fc treated R6/2 and vehicle treated R6/2: *p < 0.05; **p < 0.01; ***p < 0.001. n = 11 WT vehicle (EDL), n = 12 WT vehicle (TA), n = 8 WT ACVR2B/Fc (EDL and TA), n = 14 R6/2 vehicle (EDL and TA), n = 12 R6/2 ACVR2B/Fc (EDL and TA). All data presented as means ± SEM. The baseline WT and R6/2 vehicle treated phenotype data were previously published^10^.

Neuromuscular function was assessed for the extensor digitorum longus (EDL) and TA muscles after treatment of female mice with either vehicle or ACVR2B/Fc at 12 weeks of age as compared to their littermate controls. We began by measuring the isometric muscle tension of these two muscles to assess the effect of ACVR2B/Fc on the contractile abnormalities that we have previously described in R6/2 mice^19,22^. EDL is normally a fast twitch muscle that contracts and relaxes rapidly. In R6/2 mice, EDL took longer than WT EDL to exert a maximum twitch and then longer to relax (Fig. 3A and C). Upon treatment, the time to reach maximum twitch force was restored (Fig. 3A). In R6/2 mice TA muscles take the equivalent time as WT TA muscles to reach the peak force but take longer to relax (Fig. 3B and D). ActRIIB treatment completely restored the contractile dysfunction in the R6/2 TA (Fig. 3D).

Next, we measured the maximum tetanic force exhibited by the EDL and TA muscles in ACVR2B/Fc and vehicle treated WT and R6/2 mice. The EDL and TA muscle of R6/2 mice were weaker as compared to those from WT littermates (Fig. 3E and F); ACVR2B/Fc treatment increased R6/2 EDL strength to WT levels and there was a slight increase in the strength of the R6/2 TA muscles (87.2 ± 8.2 g as compared to 68.7 ± 4.5 g, p < 0.1) (Fig. 3E and F).

EDL muscles in WT mice are innervated by approximately 30 functional motor units^35^ and we have previously shown that this is reduced to approximately 20 motor units in the R6/2 EDL at 12 weeks of age and then further decreased to approximately 10 units by 14 weeks^19^. The number of functionally active motor neurons that innervate the EDL was assessed in vehicle and ACVR2B/Fc treated 12 week old WT and R6/2 mice. Representative examples of motor unit recordings and the mean motor unit number are illustrated in Fig. 3G. ACVR2B/Fc treatment completely prevented the loss of functional motor units in R6/2 mice and had no effect on motor unit number in WT mice.

### Treatment with ACVR2B/Fc delays end-stage disease but does not improve motor impairments

**Figure 4:**
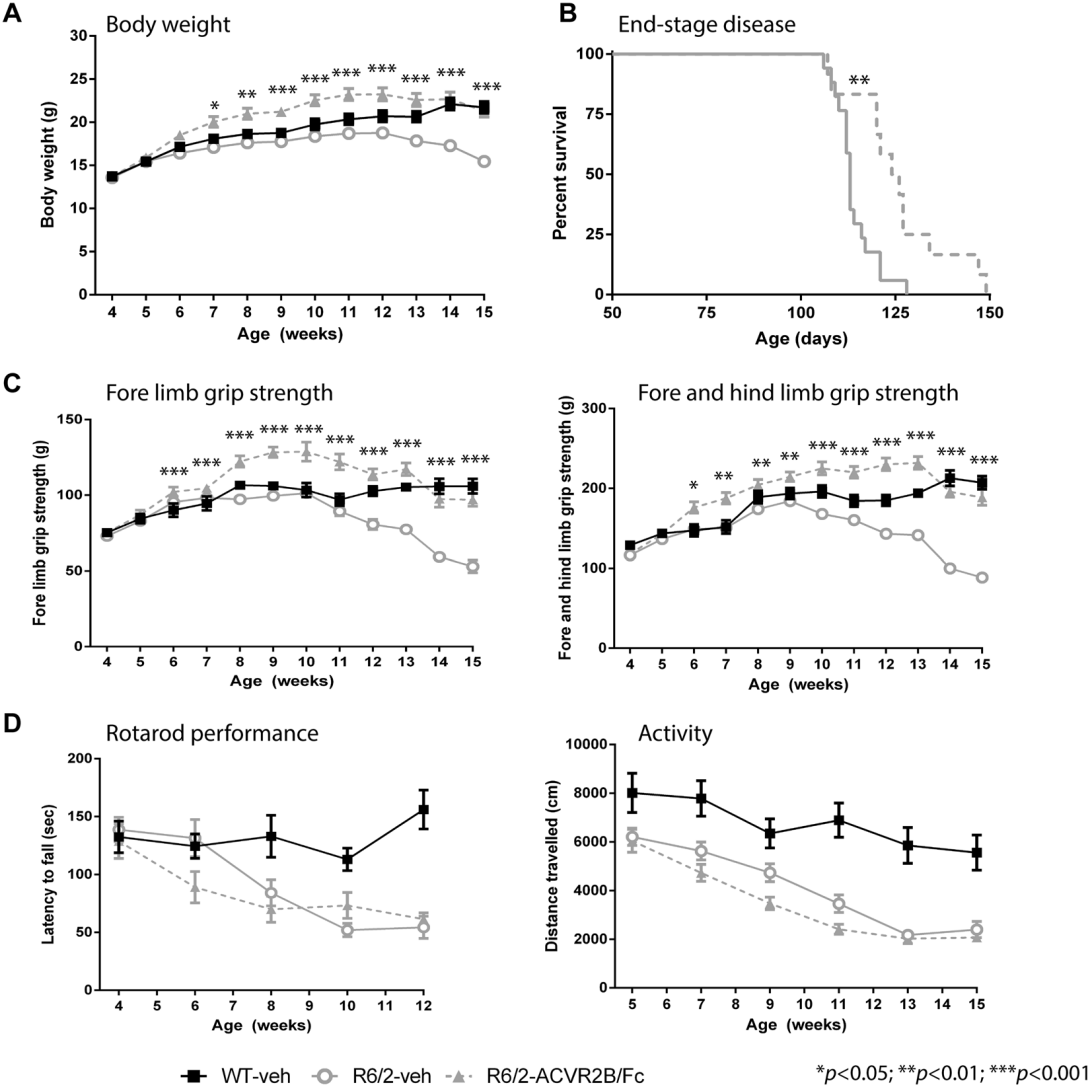
Treatment with ACVR2B/Fc delays end-stage disease, but has no effect on rotarod performance or activity measures. (**A**) ACVR2B/Fc treatment completely prevented the progressive loss in body weight loss that occurs in R6/2 mice. The effect was such that R6/2 mice were significantly heavier than WT mice at some ages (Table S9). (**B**) Kaplan-Meier curve showing that ACVR2B/Fc treatment delays end-stage disease in R6/2 mice (Chi = 8.764, *p* < 0.01 Mantel-Cox log-rank test). (**C**) ACVR2B/Fc treatment completely prevented the progressive loss in fore-limb as well as the combined fore- and hind-limb grip strength that occurs in R6/2 mice. The effect was such that R6/2 mice were significantly stronger than WT mice at some ages (Table S9) (**D**) ACVR2B/Fc had no effect on the impairment in R6/2 rotarod performance or hypoactivity. Statistical analysis was two-way ANOVA with post-hoc Bonferroni correction (see Table S8 for main effects and Table S9 for multiple comparisons). The statistical significance between values for ACVR2B/Fc treated and vehicle treated R6/2 mice is depicted: *p < 0.05; **p < 0.01; ***p < 0.001. WT vehicle, n = 13; R6/2 vehicle, n = 17; R6/2 ACVR2B/Fc, n = 14. All data presented as means ± SEM.

The progressive impairment of motor function is a cardinal feature of HD mouse models, to which both central nervous system (CNS)-driven and skeletal muscle-driven pathogenic mechanisms might contribute. Given that ACVR2B/Fc treatment completely rescued muscle atrophy and weakness, this provided an opportunity to dissect the extent to which a muscle-based pathology might contribute to motor related tasks. To address this, we dosed female R6/2 mice with either vehicle or ACVR2B/Fc from five weeks of age. A vehicle treated WT group was included to allow an estimation of effect size and to provide a control for any adverse effects (Fig. 4). Body weight and fore limb and hind limb grip strength were measured until 15 weeks of age. Mice were assessed for performance on an accelerating rotarod until 12 weeks of age and activity measures until 15 weeks. As before, ACVR2B/Fc treatment rescued deficits in body weight (Fig. 4A), and this rescue was maintained until 15 weeks of age. End-stage disease, as defined by the criteria in Table S2, was statistically significantly delayed in treated mice (Fig. 4B). Due to the development of an anal prolapse, two ACVR2B/Fc treated R6/2 mice were culled (on days 106 and 122) and these were not included in the final analysis. Although the deficits in grip strength were completely restored (Fig. 4C), there was no improvement in the decline in rotarod performance or the ensuing hypoactivity in the open field (Fig. 4D). Therefore, the restoration in muscle mass and strength did not improve the motor-related tasks measured here, suggesting that the pathogenic basis of these impairments is driven by CNS-related mechanisms.

### ACVR2B/Fc treatment and huntingtin aggregation

HTT aggregation in the form of nuclear inclusions has been detected in the skeletal muscle of both R6/2 and the *Hdh*150 knock-in HD models^13,20,21^. Quantification of the aggregate load using the Seprion ELISA has demonstrated that statistically significant levels of aggregated HTT can be detected in R6/2 quadriceps by 8 weeks of age^36^ and that the aggregate load is higher in the TA than in the quadriceps or gastrocnemius/plantaris^19^.

**Figure 5:**
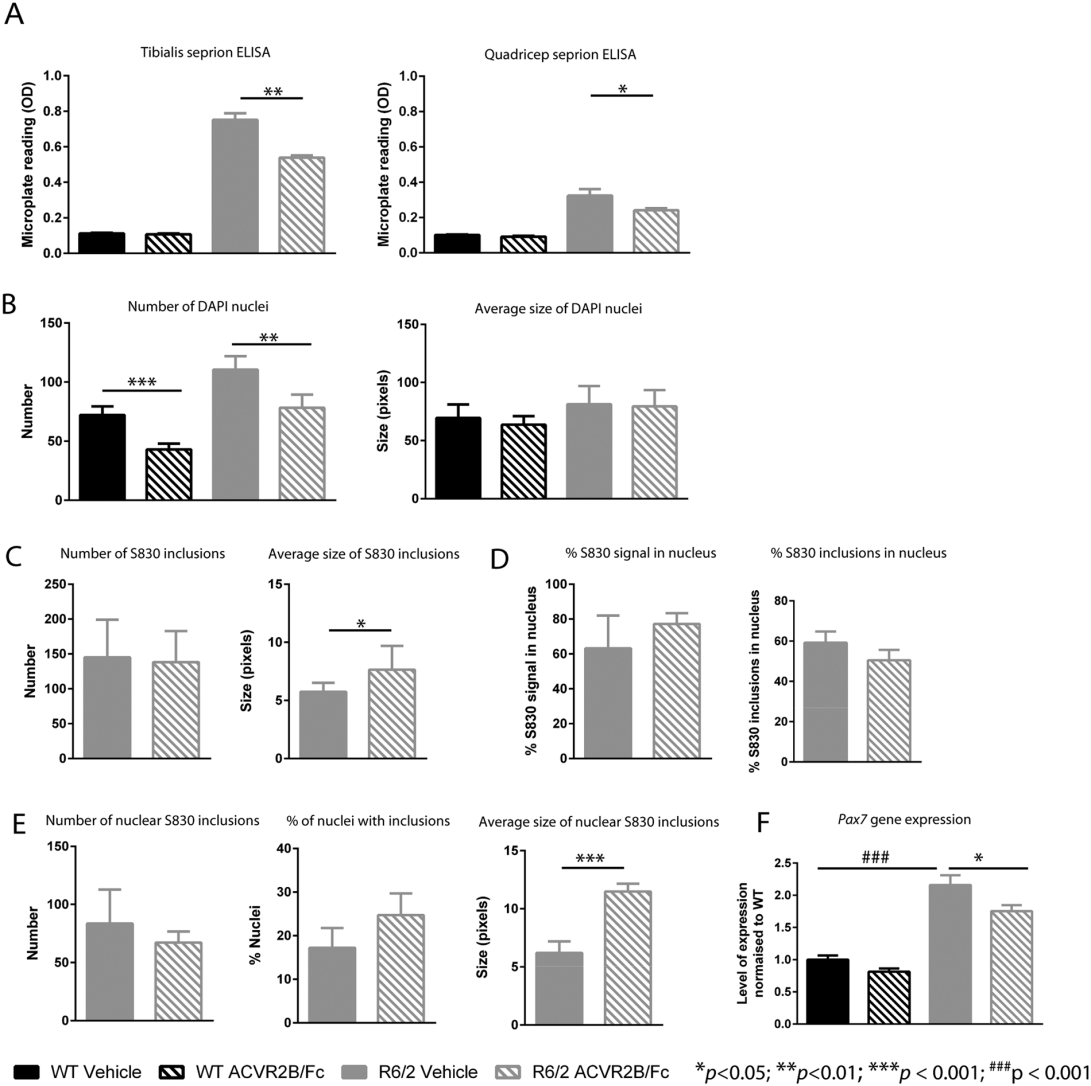
ACVR2B/Fc treatment and HTT aggregation. (**A**) ACVR2B/Fc treatment results in a decrease in the aggregate load in R6/2 TA and quadriceps muscles as assessed by the Seprion ELISA. WT vehicle, n = 5; WT ACVR2B/Fc, n = 10; R6/2 vehicle n = 10; R6/2 ACVR2B/Fc, n = 9. WT background signal derives from the substrate. (**B**) Number of DAPI stained nuclei per ROI and average nucleus size in DAPI pixels. (**C**) Number of S830 inclusions per ROI and average inclusion size in pixels. (**D**) Percentage of S830 signal co-localized with DAPI and percentage of inclusions localized to the nucleus. (**E**) Number of nuclear S830 inclusions per ROI, the percentage of nuclei with inclusions and average size of nuclear inclusions in pixels (**F**) Level of expression of *Pax7* as fold change from WT. Taqman qPCR values were normalized to the geometric mean of *Atp5b*, *Actb* and *Sdha*. WT vehicle, n = 8; WT ACVR2B/Fc, n = 7; R6/2 vehicle n = 6; R6/2 ACVR2B/Fc, n = 8. Statistical analysis for (**B**) and (**D**) was two-way ANOVA with post-hoc Bonferroni correction (see Table S8 for main effects) and for (**A**), (**C**), (**D**) and (**E**) was Student’s *t*-test (n = 4/treatment group). Statistical significance for R6/2 vehicle vs R6/2 ACVR2B/Fc is depicted by *p < 0.05; **p < 0.01; ***p < 0.001 and for WT vehicle vs R6/2 vehicle by ^###^p < 0.001. All data presented ± SEM. WT = wild type, ROI = regions of interest.

We began by applying the Seprion ELISA to investigate the effect of ACVR2B/Fc treatment on HTT aggregation in R6/2 quadriceps and TA muscles from 12 week old mice that had been treated with ACVR2B/Fc or vehicle. This suggested that treatment had led to a reduction in the aggregate load in both TA (*p* < 0.01) and quadriceps (*p* = 0.05) (Fig. 5A). HTT aggregation is the earliest phenotype to have been detected in skeletal muscle, and therefore, this result suggested that ACVR2B/Fc treatment might have had disease modifying properties. However, alternatively, the ACVR2B/Fc induced hypertrophy might have diluted the level of HTT aggregation in a given tissue mass, in which case, the degree of HTT aggregation could have remained unchanged, and our Seprion ELISA results might have been misleading. To investigate this further, TA sections were immunostained with antibodies to HTT (S830), and laminin, counterstained with DAPI (Fig. S2A and C), and immunofluorescence signals were quantified as illustrated in the work flow in Fig. S2B. The number of DAPI-stained nuclei in the regions of interest (ROI) was increased in R6/2 TA as compared with WT, which is consistent with the myofibre atrophy that had occurred in this R6/2 muscle (Figs 5B and S3A). Conversely, the number of nuclei had decreased in both WT and R6/2 TA in response to ACVR2B/Fc treatment, consistent with the hypertrophic response (Figs 5B and S3A). The average size of the nuclei, as determined by DAPI pixels, was unchanged between WT and R6/2 TA and did not change upon treatment in either case (Figs 5B and S3A).

To investigate the pattern and level of aggregation we quantified the level of S830 immunofluorescence. There were an equivalent number of S830 reactive inclusions in the ROI in R6/2 TA muscle that had been treated with either vehicle or with ACVR2B/Fc (Fig. 5C) although the average size of the inclusions (as measured in red pixels) was greater in the ACVR2B treated muscle (Fig. 5C). Previous studies have documented the formation of nuclear inclusions in the skeletal muscle of HD mouse models^13,20^, however, our immunostaining clearly demonstrated that cytoplasmic inclusions were also present (Fig. S2C). To compare the level of aggregated HTT between the nucleus and cytoplasm, we performed an unbiased examination of the subcellular localization of the aggregates in the vehicle and ACVR2B/Fc treated R6/2 TA muscle. Approximately 70% of the S830 immunoreactivity co-localized with DAPI staining (Figs 5D and S3B) for both treatment groups, corresponding to approximately 55% of inclusions occurring in the nucleus (Fig. 5D). This is consistent with our observation that the nuclear inclusions tended to be larger than those in the cytoplasm.

We next examined the frequency and size of nuclear inclusions between R6/2 TA muscle that had been treated with ACVR2B/Fc and vehicle. Given that the average size of all inclusions per ROI was larger in ACVR2B/Fc treated muscle (Fig. 5C), that the total level of S830 signal in the nucleus was equivalent between treatment groups (Figs 5D and S3C) and that there were fewer nuclei in ACVR2B/Fc treated muscle (Fig. 5B), we asked whether the nuclear inclusions were larger in the ACVR2B/Fc treated TA. We found that the number of inclusions that co-localised with the DAPI staining was equivalent between the two treatment groups (Fig. 5E); that there was a trend toward an increase in the percentage of nuclei containing inclusions in the ACVR2B/Fc treated TA (*p* = 0.097) (Fig. 5E) and that the size of the nuclear inclusions was greater in the ACVR2B/Fc treated TA (Fig. 5E). These data are consistent with the ACVR2B/Fc treated TA having fewer nuclei per ROI (Fig. 5B), of which a higher percentage have larger inclusions than in the vehicle treated TA.

Skeletal muscle regenerates in response to injury and *Pax7* expressing satellite cells are the major or only mediators of myofibre regeneration in the adult mouse^37^. After multiple rounds of injury, the satellite pool is maintained through a process of self-renewal^37^. We found an increased level of *Pax7* expression in R6/2 TA as compared to WT at 12 weeks of age suggesting that the satellite cell pool comprises a greater percentage of total nuclei in R6/2 than in WT mice. Treatment with ACVR2B/Fc brought the level of *Pax7* expression back toward WT levels, indicating that the percentage of nuclei expressing *Pax7* is lower in ACVR2B/Fc treated than in vehicle treated R6/2 mice (Fig. 5F).

### ACVR2B/Fc activates a transcriptional program to increase muscle mass, with little impact on HD-related transcriptional dysregulation

Transcriptional dysregulation is a well-established pathogenic process in HD^38^. Microarray profiles have been used to identify dysregulated transcripts in specific regions of HD *post mortem* brains^39,40^ and in quadricep biopsies from HD patients^23^ as compared to control subjects. Given that the transcriptome is highly dysregulated in the brain^12^ and skeletal muscle^23^ of R6/2 transgenic mice, we used quantitative real time PCR (qPCR) to investigate the level of expression the ActRIIB receptor (*Acvr2b*) and myostatin (*Mstn*), and found that the receptor and ligand were expressed at comparable levels in WT and R6/2 muscle, hence their transcriptional dysregulation does not contribute to the muscle wasting in R6/2 mice (Fig. S4). The expression levels of *Mstn* and *Acvr2b* were not affected by treatment in either case.

Inhibition of myostatin signaling activates pathways that directly stimulate protein synthesis and inhibit protein breakdown in the muscle fibre^32^. In order to uncover the transcriptional changes that underlie the phenotypic rescue by ACVR2B/Fc, RNA from the quadriceps and TA of WT and R6/2 mice at 12 weeks of age that had been treated with either vehicle or ACVR2B/Fc was sequenced (n = 10/treatment group). Dysregulated gene analysis (DESeq. 2) was applied to identify genes for which there was a statistically significant change in expression (Benjamini-Hochberg adjusted *p* < 0.05). The transcriptional response of WT and R6/2 muscles to ACVR2B/Fc treatment was relatively comparable (Table S3). We did not see changes in the expression of genes controlled by SMAD 2/3-signaling. This is most likely because the RNAseq analysis was performed on tissue harvested one week after the last ACVR2B/Fc dose, by which time this response had diminished. The gene ontology (GO) terms associated with the few hundred transcripts whose expression levels were altered in response to treatment, in both WT and R6/2 mice, were broadly related to muscle function and energy metabolism (Tables S3 and S4).

**Figure 6:**
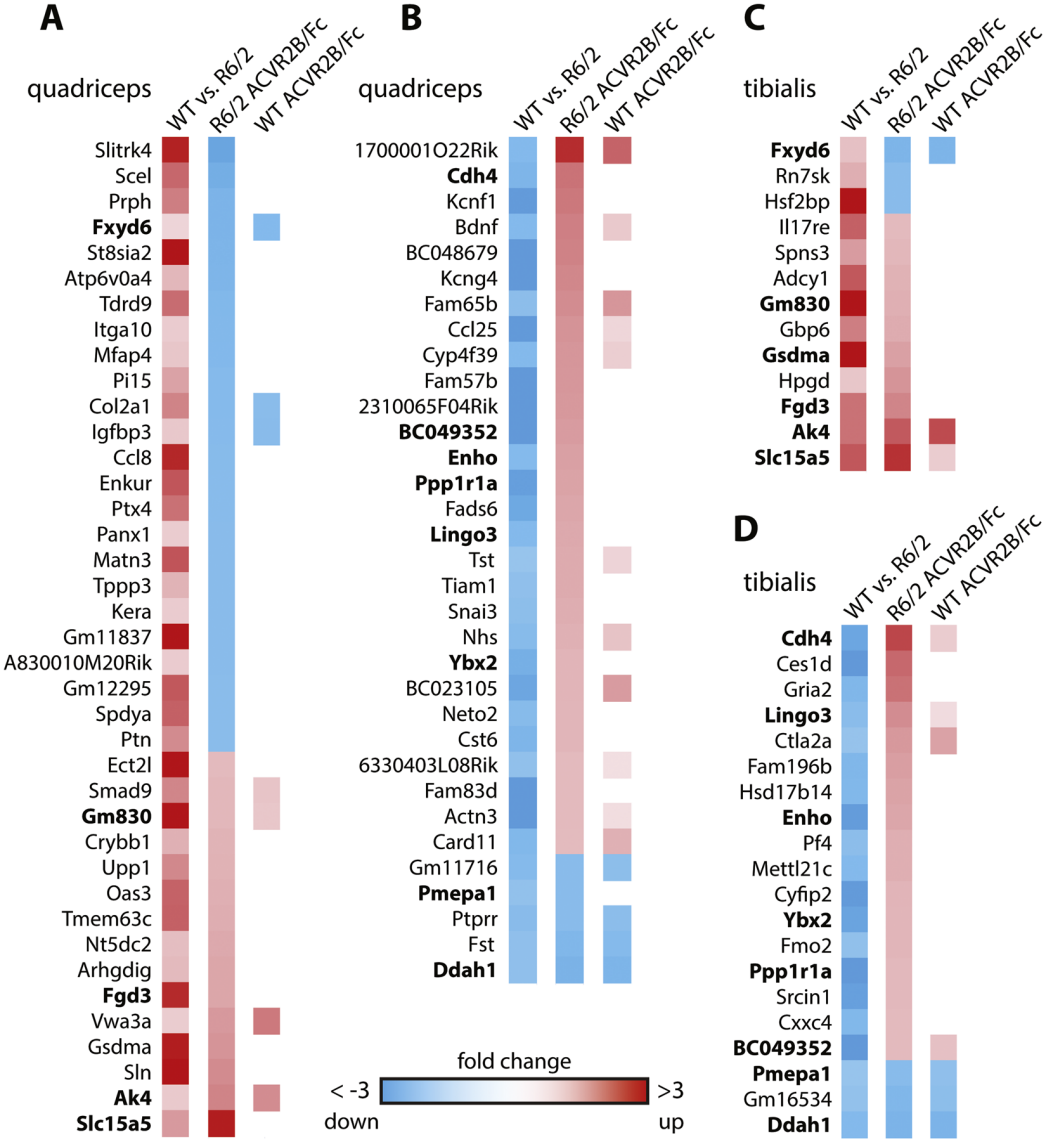
The effect of ACVR2B/Fc treatment on genes that are dysregulated in R6/2 muscle. The first column in each panel indicates the change in gene expression in R6/2 muscle as compared to WT. Red squares represent an increase and blue squares, a decrease in expression levels. The second column represents the change in gene expression levels in R6/2 muscle in response to ACVR2B/Fc treatment and the third column indicates when a gene in WT muscle was also significantly changed in response to treatment. All of the depicted genes were changed to statistically significant levels (adjusted *p* < 0.05) with a fold change greater than 1.5. Genes in bold were changed in both quadriceps and tibialis anterior. The panels represent genes that are (**A**) increased or (**B**) decreased in the quadriceps and (**C**) increased or (**D**) decreased in the tibialis anterior of R6/2 mice as compared to WT. RNAseq was performed on RNA extracted from n = 10 mice/treatment group.

The comparison of gene expression levels in the quadriceps and TA of R6/2 as compared to WT mice indicated that approximately a half of all genes were dysregulated in these muscles (Table S3). We next analysed the effect of ACVR2B/Fc treatment on the extent of transcriptional dysregulation in R6/2 TA and quadriceps. We found that the expression level of only a very small number of R6/2-dysregulated genes was changed (cut off of 1.5 fold change and adjusted to *p* < 0.05) (detailed in Fig. 6 and Table S4). In some cases, administration of ACVR2B/Fc restored the expression levels of dysregulated genes toward WT levels, but in others, the extent to which genes were dysregulated was increased (Fig. 6, Table S4). Therefore, inhibition of signaling through the ActRIIB receptor had very little impact on the disease-related transcriptional dysregulation in these two muscles. We applied weighted gene correlation network analysis (WGCNA), in an attempt to uncover subtle changes in transcriptional regulation, which might not have been picked up by DESeq. 2, however, this did not reveal any additional GO terms (Tables S5 and S6).

## Discussion

Pathophysiological alterations in skeletal muscle contribute to the phenotypes exhibited by mouse models of HD (for review see^28^). Here we have shown that treatment with a soluble ActRIIB receptor (ACVR2B/Fc) completely prevented skeletal muscle atrophy and body weight loss from occurring in the R6/2 HD mouse model. Treatment prevented the onset of skeletal muscle weakness, contractile abnormalities and the loss of functional motor units in EDL muscles. Remarkably, ACVR2B/Fc treatment also delayed the age at which R6/2 mice reached end-stage disease.

The deposition of aggregated forms of the HTT protein is the earliest phenotype to have been reported in the skeletal muscle of HD mouse models^36^, and therefore it was important to determine whether treatment with ACVR2B/Fc might have modulated this process, which would potentially be indicative of disease modifying properties. However, our assessment of the comparative levels of HTT aggregation turned out to be more complicated than anticipated. Our routine approach to measuring HTT aggregation, using the Seprion ELISA, suggested that ACVR2B/Fc treatment might have decreased the HTT aggregate load. However, this interpretation was complicated by two factors: the ACVR2B/Fc induced hypertrophy resulted in a lower density of nuclei in the treated muscle, potentially diluting the levels of both nuclear and cytoplasmic aggregates; second, the satellite cell pool was greater in vehicle than in ACVR2B/Fc treated muscle reflecting attempts by the R6/2 muscle at regeneration in response to mutation-induced pathogenic processes. In fact, our immunohistochemical analyses indicated that the levels of aggregation in the vehicle and ACVR2B/Fc treated muscles were relatively comparable, although the size and frequency of nuclear inclusions differed. The ACVR2B/Fc treated muscle had a lower density of nuclei, of which a higher percentage contained larger nuclear inclusions than in the vehicle treated samples. It is possible that replenishment of the satellite cell pool in vehicle-treated R6/2 muscle provided a source of ‘younger’ nuclei in which the aggregation process was less well advanced. A more detailed analysis of the effect of ACVR2B/Fc on HTT aggregation requires a much better baseline understanding of this process in skeletal muscle and is beyond the scope of this study at the present time.

Inhibition of myostatin/activin A signaling activated a relatively comparable transcriptional program in both WT and R6/2 mice to increase muscle protein synthesis and decrease the degradation of muscle proteins. The gene ontology terms associated with these changes were broadly associated with muscle function and energetics. Our RNAseq transcriptome analysis showed that approximately one half of all genes were dysregulated in the R6/2 TA and quadriceps at 12 weeks of age as compared to wild type. In light of the complete restoration of muscle mass and strength, it was surprising that we found such little evidence of an improvement in this HD-associated transcriptional dysregulation. However, this result was compatible with the fact that ACVR2B/Fc treatment did not prevent HTT aggregation in nuclei.

The mouse models in which skeletal muscle phenotypes have been described all carry highly expanded CAG repeats and represent the severe forms of HD with symptom onset occurring in childhood or adolescence. Therefore, is there any evidence that these preclinical findings will be applicable to HD patients with CAG repeat expansions that cause the adult onset form of the disease? Although studies are limited, impairments in skeletal muscle function have been reported in this patient group. Muscle strength has only been assessed in one study. This used a hand-held dynamometer to measure the isometric muscle strength in six lower limb muscle groups and found that people with HD had, on average, half the strength of healthy matched controls and that muscle strength significantly correlated with stage of disease as measured by UHDRS (unified Huntington’s disease rating scale) scores^41^. Strikingly, myopathy was reported to be the first HD-related symptom in a semi-professional marathon runner, which occurred years before the appearance of other neurological symptoms^42^. Most mechanistic studies of the skeletal muscle pathophysiology of HD patients have focused on mitochondrial function and energy metabolism. These have revealed a decreased oxidative capacity^43,44^ and lower anaerobic threshold^45^ than in control subjects and deficits in complex II/III activity of mitochondrial respiratory chain^46^, although normal mitochondrial function has recently been reported in presymptomatic mutation carriers^47^. Gene expression profiling of the RNA from HD patient skeletal muscle biopsies revealed the beginnings of a transition from fast twitch to slow twitch fibres^23^, consistent with that observed in the mouse models^19,23^. Histological studies have been very limited, often being restricted to the analysis of a single patient, and have reported: multiple centralized nuclei^44^, non-specific myopathic changes^48^, an abnormal pattern of HTT staining^44^ and the presence of nuclear inclusions^46^. A systematic longitudinal study of muscle function and pathology at all clinical stages of HD is clearly warranted.

In general, systematic muscle wasting is associated with weakness, fatigue, frailty, insulin resistance, bone fracture, disability and death^32^. In HD, muscle atrophy and weakness would be expected to exacerbate physical inactivity, which could in turn increase the rate of atrophy^41^. It may contribute to the frequency of falls and accelerate the loss of independent mobility and the use of a wheelchair. It could also lead to an increase in HD-associated apathy thereby contributing to cognitive decline. Myostatin inhibition in muscle has been shown to improve metabolism, effects that may also be beneficial to individuals with HD^49^. Therefore, although our data suggest that inhibition of myostatin/activin A signalling would provide a symptomatic treatment rather than one targeting the underlying pathogenic basis of the disease, this may have considerable impact on the overall quality of life for HD patients in terms of increased mobility and a reduction in falls, as well as the cognitive and mood benefits that could arise through an increased exercise capacity^50,51^. Consistent with this approach, myostatin inhibitors are currently being tested in clinical trials as a symptomatic treatment for inclusion body myositis^34,52^ and have been proposed as treatments to target the periphery in amyotrophic lateral sclerosis^53^.

The development of agents that inhibit myostatin signalling has been an active area of research since the knock-out of myostatin was shown to induce muscle hypertrophy in mice^30^. These approaches fall into two main categories, those that specifically target myostatin e.g. anti-myostatin antibodies, or those that, like the ACVR2B/Fc soluble receptor used here, also inhibit signaling via its alternative ligands: activin A, growth differentiation factor 11 (GDF11) and bone morphogenic proteins (BMPs)^32^. Therapeutics that inhibit myostatin/activin A signaling are currently undergoing clinical trials for a wide range of indications which include sarcopenia, cancer cachexia, muscular dystrophy, sporadic inclusion body myositis and rehabilitation post-orthopedic surgery (for reviews, see^34,54^). The comparative safety, tolerability and efficacy of these modalities will soon have been established^34^ paving the way to the design of clinical trials to assess the benefits of myostatin inhibition in individuals with HD.

## Methods

### Mouse maintenance, breeding and genotyping

All animal care and procedures were performed in compliance with the regulation on the use of Animals in Research (UK Animals and Scientific Procedures Act of 1996 and the EU Directive of 2010/63/EU) with approval by the King’s College London Ethical Review Process Committee. Hemizygous R6/2 mice were bred by backcrossing R6/2 males to (CBA x C57BL/6) F1 females (B6CBAF1/OlaHsd, Harlan Olac, Bicester, UK). All animals had unlimited access to water and breeding chow (Special Diet Services, Witham, UK), and housing conditions and environmental enrichment were as previously described^55^. Mice were subject to a 12-h light/dark cycle. Genotyping of tail DNA by PCR and measurement of the CAG repeat length were performed as previously described^36^. Dissected tissues were weighed before being snap frozen in liquid nitrogen for molecular analyses and stored at −80 °C until further analysis.

### ACRB2R/Fc treatment

ACVR2B/Fc was prepared as previously described^56^ in a series of batches and stored at 4 °C in PBS as vehicle. It was administered weekly by subcutaneous injection at 10 mg/kg starting at 5 weeks of age. For most experiments, the last dose was given at 11 weeks of age and mice were sacrificed one week later at 12 weeks. Mice were randomized to treatment groups based on body weight, litter of origin and CAG repeat size and investigators were blind to genotype and treatment group throughout each trial. Body weight and/or grip strength were measured longitudinally for all experiments to ensure that all batches of ACVR2B/Fc had comparable efficacious effects (Table S7). A reduction in body temperature can be indicative of an adverse response to a treatment. Body temperature was monitored weekly using an infra-red temperature reader (ThermoScan Instant Thermometer, Braun) that was reproducibly positioned under the thorax. There was no change in body temperature (Fig. S1B), which taken together with other measures of appearance, indicated that the treatment was well-tolerated. For the assessment of ACVR2B/Fc treatment on end-stage disease, dosing continued weekly after 11 weeks of age until it was judged that a mouse had reached its humane end-point (Table S2). A summary of the mice used in each experiment and their CAG repeat size is listed in Table S7.

### Assessment of motor-related tasks

Grip strength was measured as previously described^57^. For the initial pilot study, fore-limb grip strength was measured using a San Diego Instruments Grip Strength Meter (San Diego, CA, USA) and for subsequent experiments, fore-limb grip strength and also the combined fore- and hind-limb grip strength were measured using a grip strength meter from Bioseb *in Vivo* Research Instruments. Mice were tested for motor co-ordination as previously described^58^ on an Ugo Basile accelerating rotarod (Linton Instruments, UK) that had been modified with a smooth rubber coating over the beam^55^. General ambulation was probed in square, plain white arenas (50 × 50 × 50 cm, Engineering Design Plastics Ltd, Cambridge, UK) for 30 min and behaviour was recorded through a video camera positioned above the apparatus. Activity (distance moved) was tracked and then analysed using Ethovision 7XT software (Noldus, Netherlands) as described^57^.

### Assessment of muscle function *in vivo*

Twelve week old female WT and R6/2 mice that had been treated with vehicle or ACVR2B/Fc were deeply anesthetized with isoflurane. The distal tendons of the tibialis anterior (TA) and extensor digitorum longus (EDL) muscles in both hindlimbs were dissected free and the isometric tension was recorded *in vivo* as described in detail elsewhere^35^. The contractile characteristics were determined by measuring the time taken (ms) for the muscle to elicit peak twitch tension (time to peak, TTP) and the time taken for the muscle to reach half relaxation from peak contraction (half relaxation time). Tetanic contractions were measured as previously described^35^ and recorded on a Lectromed Multitrace 2 recorder (Lectromed Ltd, UK). All parameters were measured using the Picoscope v5 and v6 software (Pico Technology, Cambridgeshire, UK). The number of functional motor units innervating the EDL muscles was determined as described previously^35^.

### Immunohistochemistry and lesser fibre diameter measurements

Muscles were dissected, embedded, frozen in isopentane and sectioned as previously described^59^. Immunohistochemical staining was performed using the Ventana Discovery XT instrument, using the Ventana DAB Map Kit (760–124). Anti-laminin (Sigma L9393) primary antibody incubation was for 1 h using a 1:1000 dilution. Swine anti- Rabbit (Dako E0353) secondary antibody incubation was for 32 min, using a 1:200 dilution. Slides were haematoxylin counterstained.

Analysis of laminin stained muscle sections was performed on whole slide images, generated using a Leica SCN400F, using Definiens Developer XD (Definiens, Munich, Germany) and between 3 and 8 tissue sections were analysed per mouse. An automatic threshold method was used to separate the tissue from the background area, then again to identify sarcolemma within the tissue. A series of shape based manipulations and edge smoothing operations were then performed to separate joined fibres, combine split fibres and clarify the definition of muscle fibres. Finally muscle fibres which do not satisfy strict morphometric rules were excluded to provide a reliable set of transversely sectioned muscle fibres; width and shape measurements were then exported. The fibre diameter profile each tissue section was generated in excel.

### MR Imaging and sample preparation

The methods for sample preparation, scanning and data processing are described in detail in the Supplemental Information.

### HTT aggregation analysis by ELISA and immunohistochemistry

Aggregates were captured in Seprion ligand coated plates (Microsens, PADPCB1 SEP1–01) and detected using the MW8 mouse monoclonal antibody^60^ (1:2000) as previously documented^36^. For immunohistochemistry, muscles were dissected, embedded, frozen in isopentane and sectioned as described^59^ and slides were stored −20 °C until processing for immunohistochemistry. Prior to staining, slides were equilibrated to 50 °C to remove moisture and a wax pen was used to create a hydrophobic barrier around the sections. After washing with PBS, sections were blocked using 10% normal donkey serum and 0.3% triton X-100 in PBS. Antibodies against laminin (Sigma L9393, 1:1000) and HTT (S830^61^, 1:1000) were applied overnight at 4 °C prior to the application of appropriate secondary antibodies (Molecular Probes, 1:500) at RT for 2 h followed by the DAPI nuclear stain for 15 min.

Confocal focal images were taken using the Nikon AR1 Confocal microscope (Nikon Instruments using a 40x objective (Plan Apo 40x Ph2, NA 0.95) and NIS-Elements C software (Nikon Instruments). A grid was applied to capture nine regions of interest (ROI) per section through an automated unbiased process from two muscle sections per mouse (n = 4 mice per treatment group) to generate 18 captured ROIs per mouse. Images were exported as TIFFs and analysed using threshold fluorescence levels in ImageJ (U. S. National Institutes of Health, http://imagej.nih.gov/ij/) (Fig. S2A and C). A threshold intensity value of 90 was applied to DAPI images and of 50 to S830 images, and pixels below these thresholds were excluded. The S830 signal in the sections from WT mice treated with either vehicle or with ACVR2B/Fc was negligible (Fig. S3C). Objects were identified as groups of adjacent pixels. Objects with less than 25 DAPI pixels were considered debris and not counted as nuclei. DAPI threshold images were used to mask the S830 images and co-localised pixels were counted as intra-nuclear inclusions and those not co-localising were considered to be extra-nuclear inclusions (Fig. S3B). The percentage of nuclei containing inclusions was counted manually.

### RNA extraction and Taqman real-time PCR expression analysis

Total RNA from skeletal muscles was extracted with the mini-RNA kit according to the manufacturer instructions (Qiagen). The reverse transcription reaction (RT) was performed using MMLV superscript reverse transcriptase (Invitrogen) and random hexamers (Operon) as described elsewhere^19,62^. The final RT reaction was diluted 10-fold in nuclease free water (Sigma). All Taqman qPCR reactions were performed as described previously^62^ using the CFX96 Real-Time PCR Detector (BioRad). Stable housekeeping genes for qPCR profiling of various skeletal muscles for HD mouse models were determined using the Primer Design *geNorm Housekeeping Gene Selection Mouse Kit with PerfectProbe* software. Estimation of mRNA copy number was determined in triplicate for each RNA sample by comparison to the geometric mean of three endogenous housekeeping genes (Primer Design) as described^62^. Primer and probe sets for genes of interest were purchased from Thermo Fisher Scientific.

### RNA sequencing and data analysis

RNA was prepared from the quadriceps and tibialis from n = 10 mice per treatment group and sequencing was performed by Expression Analysis on an Illumina Hi-seq. 2000. Paired-end sequencing was obtained, 4-plexed across lanes for a minimum of 38 million 50mer paired reads per sample. Alignment and QC was conducted in Omicsoft using the OSA algorithm^63^ against the mouse genome version B38 with EMSEMBL gene models version R75. FPKMs were then calculated following standard formulas. QC assessment found 79 of 80 samples of high quality both at the RNA quality and alignment mapping levels. One sample (quadriceps mouse 93) was found to be a strong outlier by principal component analysis and omitted from the analysis. The RNAseq data have been deposited in the GEO database under the accession number GSE81367. Rounded counts to the next integer were used as input data for dysregulated gene analysis (DESeq. 2)^64^. Only genes with a count of 2 or more in at least 10 out of the 79 samples were used for the analysis which left 18558 genes in the dataset. For weighted gene co-expression network analysis (WGCNA) we used variance stabilizing transformed counts from the DESeq. 2 output (function varianceStabilizingTransformation) as input. The subsequent WGCNA analysis was carried out as previously described^40^. Soft-threshold power for all networks was 30. Modules with highly correlated module eigengenes were merged (r > 0.7). Enrichment analysis was carried out using Enrichr http://amp.pharm.mssm.edu/Enrichr/index.html ^65^. We used the gene lists of dysregulated genes or the identified network modules as input. We summarized all gene ontology terms (GO-term) of similar sub-terms into an overarching term. Only GO-terms, regulators, pathways, etc. with a combined score larger than 5 were considered.

### Statistics

Data were graphed using Prism Ver.5.0b (Graphpad software, California USA). Statistical analyses were calculated using SPSS Statistics Ver. 22 (IBM Portsmouth, UK). Data were screened for statistical outliers using Grubb’s test (GraphPad software, California, USA). The total number of mice used in each of the experiments is summarised in Table S7. The datasets can be separated into those that were collected longitudinally and those that were collected at a single time point.

#### Longitudinal datasets

These include assessment of body weight, body temperature, grip strength, locomotor activity and rotarod performance. To probe the influence of treatment across time, a two-way ANOVA was employed (Treatment group and Age as between-subject factors). All main effects from ANOVAs can be found in Table S8. Post-hoc tests with a Bonferroni correction for multiple comparisons were applied where appropriate and represented in the figures and in Table S9.

#### Datasets takes at a single time point

These include muscle mass, fibre diameters, muscle physiological measures, assessment of aggregate load by Seprion ELISA and qPCR. To probe the influence of genotype and treatment on these measures, either a two-way ANOVA was applied (Genotype and Treatment as between-subject factors) or a one way ANOVA to compare Treatment Groups. All main effects from ANOVAs can be found in Table S8 or in the text. Post-hoc tests with a Bonferroni correction for multiple comparisons were applied where appropriate and represented in the figures and in Table S9.

End-stage disease was determined using a scoring system that combined an assessment of appearance, body weight, disease score and unprovoked behaviour (Table S2). The data were represented by a Kaplan-Meier cumulative survival curve and statistical analysis was performed in SPSS using the log-rank (Mantel-Cox) test.

## Electronic supplementary material

Supplementary information
