# Supplementary material for: Myostatin inhibition prevents skeletal muscle pathophysiology in Huntington’s disease mice
Source: Sci Rep. 2017 Oct 27;7:14275. doi: 10.1038/s41598-017-14290-3 (PMC5660167; doi:10.1038/s41598-017-14290-3)
Supplement: Supplementary file 1 — Supplementary information [file 41598_2017_14290_MOESM1_ESM.pdf]

SUPPLEMENTARY INFORMATION

Myostatin inhibition prevents skeletal muscle pathophysiology in Huntington's disease mice.

Marie K. Bondulich[1,2,3]*, Nelly Jolinon[2]*, Georgina F. Osborne[1,2,3]*, Edward J. Smith[1,2,3]*, Ivan Rattray[2], Andreas Neueder[1,2,3], Kirupa Sathasivam[1,2,3], Mhoriam Ahmed[1,4], Nadira Ali[1,2,3], Agnesska C. Benjamin[1,2,3], Xiaoli Chang[5], James R. T. Dick[1,4], Matthew Ellis[6,7], Sophie A. Franklin[1,2,3], Daniel Goodwin[1,2,3], Linda Inuabasi[2], Hayley Lazell[1,2,3], Adam Lehar[5], Angela Richard-Londt[6,7], Jim Rosinski[8], Donna L. Smith[2], Tobias Wood[9], Sarah J. Tabrizi[3,7], Sebastian Brandner[6,7], Linda Greensmith[1,4], David Howland[8], Ignacio Munoz-Sanjuan[8], Se-Jin Lee[5], Gillian P. Bates[1,2,3]

METHODS

MR Imaging and sample preparation

Following treatment, at 12 weeks of age, all mice were perfuse-fixed with heparinized PBS followed by 4% paraformaldehyde (PFA; Parafix, Pioneer Research Chemical Ltd., Essex, UK) under terminal anesthesia (n = 5 / genotype / treatment). In every case, fore and hind limbs were fixed in a reproducible position. Once fixation was complete, the thoracic opening was closed using cotton sutures. The cadavers were then submerged in 4% PFA and held at 4^oC until scanning. In preparation for the scan, the fixed cadavers were removed from PFA, towel dried and submerged in Fomblin in a 50 ml falcon tube, to minimize the presence of air bubbles.

Fixed mice were scanned using a 7T pre-clinical scanner (Agilent Technologies) with a 100 Gauss gradient insert and a 39 mm quadrature birdcage RF coil (Rapid Biomedical GmbH). 3D gradient-echo images were acquired with following parameters: TR=15 ms, flip-angle 5 degrees, bandwidth 50 kHz and 3 echoes at 2.82, 3.29 and 3.76 ms. The echo times were chosen to produce fat/water signals that were in-phase, out-of-phase and in-phase again. The field of view was a 30 mm cube with a 192x192x192 matrix, for isotropic voxels of size 156.25 μm^3**.** The entirety of each mouse was imaged by repeating the scan four times, moving a new section of the sample to the isocentre of the magnet for each image. The operator ensured sufficient overlap to identify matching slices in neighboring images. Each image took 28 minutes to acquire.

For data processing, the complex-valued images were first resampled to a matrix size of 160x160x64 to improve the signal to noise ratio (SNR), resulting in an effective voxel size of 187.5x187.5x468.75 µm. The images were then separated into water and fat components using a standard 3-point Dixon technique[1]. To correct for chemical shift artifact and ensure

correspondence between water and fat images, the fat images were translated by 638 µm in the frequency-encode direction. The noise level in the fat images was calculated as the standard deviation of the signal present in a small region (32x32x32 cube) in one corner of each volume which contained no tissue. The number of voxels in each fat image that then exceeded a threshold of five times the noise floor level was counted. Overlapping slices from neighbouring volumes were identified in the water images and discarded from one volume to prevent double-counting.

Figure S1

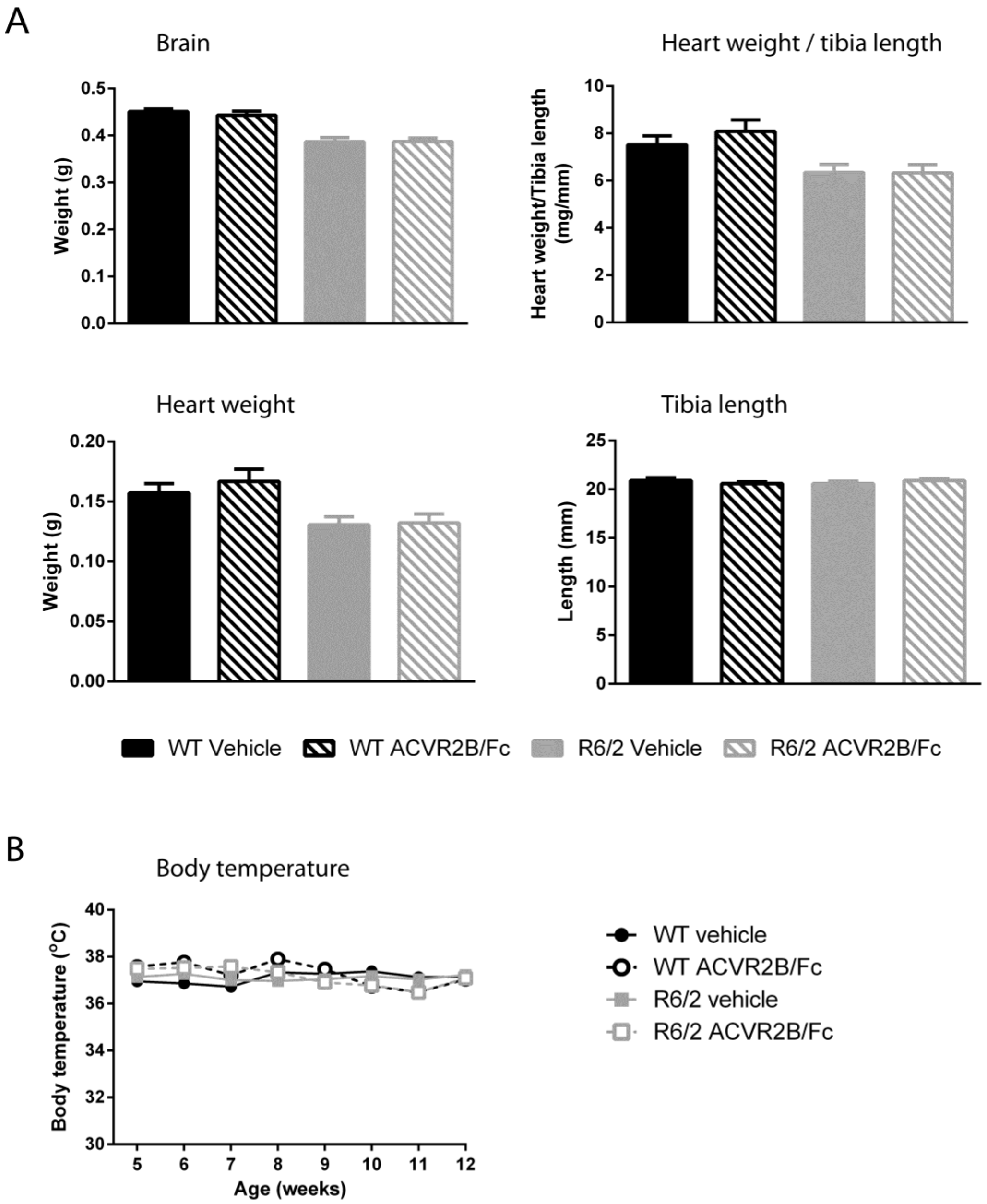

Figure S1: Treatment with ACVR2B/Fc has no effect on body temperature or heart weight in R6/2 mice. (A) ACVR2B/Fc treatment had no effect on the genotype-associated reduction in brain weight or the heart weight to tibia length index at 12 weeks of age. The heart weight and tibia length data used to calculate the heart weight / tibia length index are shown. **(B)** ACVR2B/Fc treatment has no effect on body temperature. Statistical analysis was two-way ANOVA with post-hoc Bonferroni correction (see Table S8 for main effects and Table S9 for multiple comparisons). n = 5 mice per gender per genotype. All data presented as means ± SEM.

Figure S2

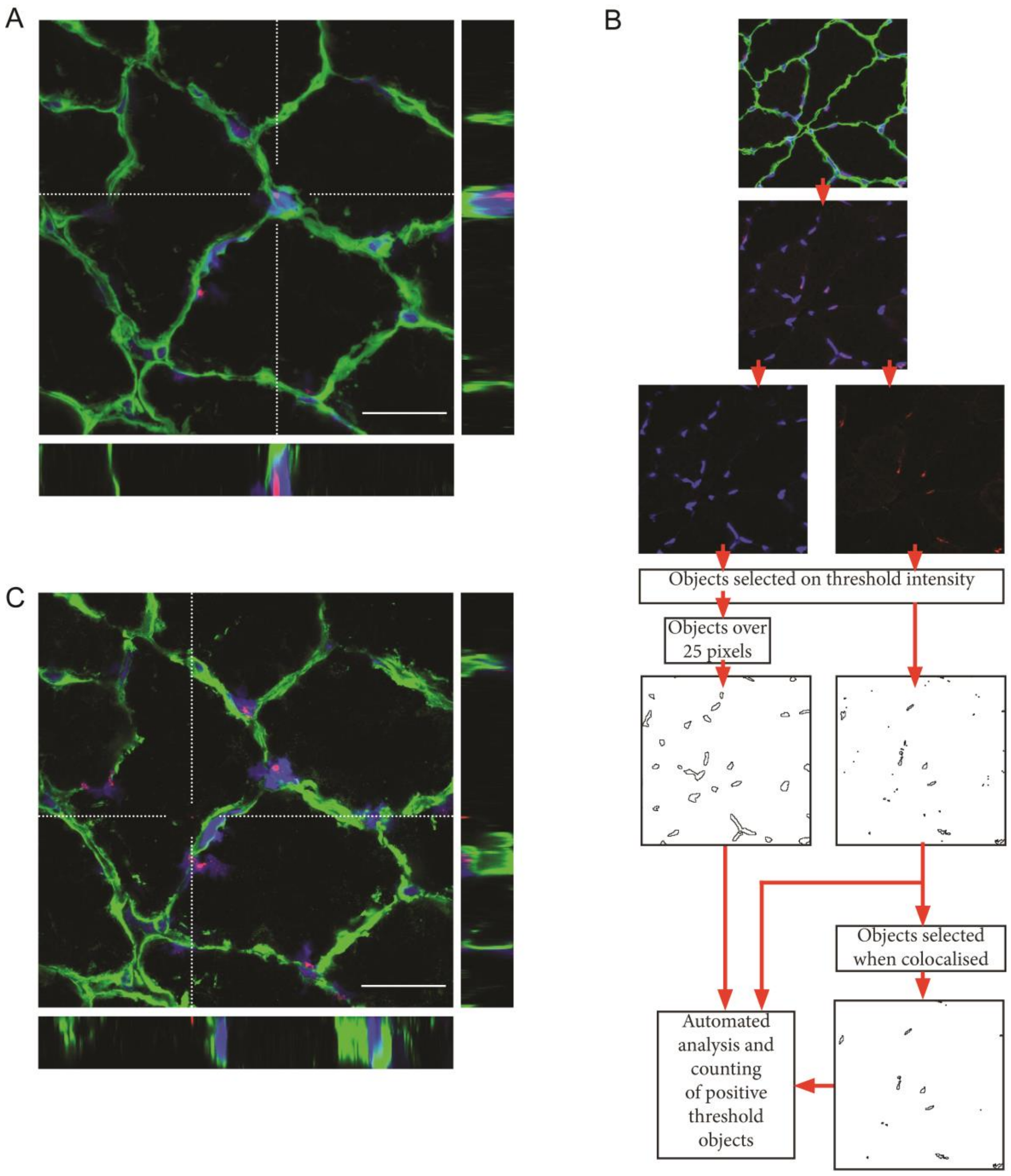

Figure S2: Confocal images of HTT aggregation in R6/2 TA muscle and analysis. (A) Confocal focal image and Z-stack showing a nuclear inclusion in R6/2 TA muscle. **(B)** A grid was applied to capture nine regions of interest (ROI) per section through an automated unbiased process from two muscle sections per mouse (n = 4 mice per treatment group) to generate 18 captured ROIs per mouse. Images were exported as TIFFs and analysed using threshold fluorescence levels in ImageJ (U. S. National Institutes of Health, http://imagej.nih.gov/ij/). A threshold intensity value of 90 was applied to DAPI images and of 50 to S830 images, and pixels below these thresholds were excluded. Objects were

identified as groups of adjacent pixels. Objects with less than 25 DAPI pixels were considered debris and not counted as nuclei. DAPI threshold images were used to mask the S830 images and co-localised pixels were counted as intra-nuclear inclusions and those not co-localising were considered to be extra-nuclear inclusions. **(C)** Confocal focal image and Z-stack showing an extra-nuclear inclusion in R6/2 TA muscle. Scale bar = 25 µm

Figure S3

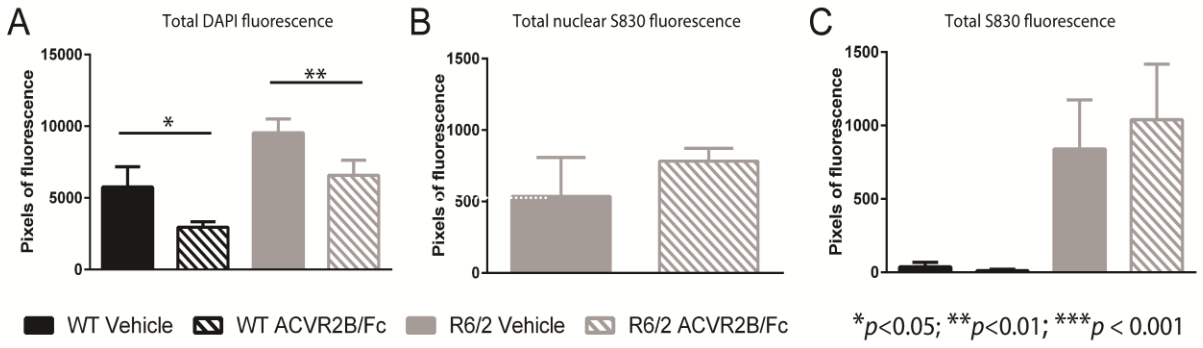

Figure S3: ACVR2B/Fc treatment and HTT aggregation. (A) Total DAPI fluorescence per ROI **(B)** Total S830 fluorescence co-localised with DAPI per ROI **(C)** Total S830 fluorescence per ROI. The background signal in the WT samples is negligible. Statistical analysis for (**A**) was two-way ANOVA with post-hoc Bonferroni correction (see Table S8 for main effects) (n = 4 / treatment group). *p < 0.05; **p < 0.01. All data presented ± SEM. WT = wild type, ROI = regions of interest

Figure S4

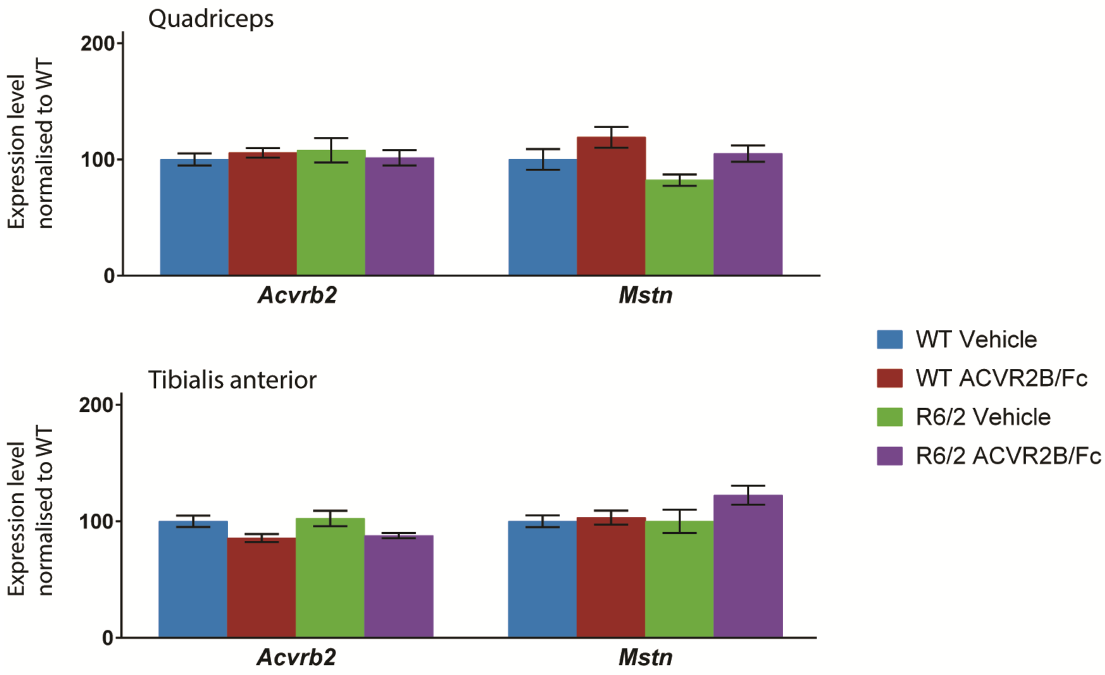

Figure S4. Expression levels of the ActRIIB receptor and myostatin genes.

The expression level of the ActRIIB receptor gene (*Acvrb2*) and myostatin (*Mstn*) were determined by qPCR and normalized to the geometric mean of the housekeeping genes: β-actin (*Actb*), ATP synthase subunit 5b (*Atp5b*) and eukaryotic translation initiation factor 4A2 (*Eif4a2*) for TA and *Actb*, *Atp5b* and *Sdha* (succinate dehydrogenase subunit A) for quadriceps. Statistical analysis was one-way ANOVA with post-hoc Bonferroni correction (see Table S9 for multiple comparisons). There was no difference in the level of expression between WT and R6/2 and this was not altered by treatment with ACVR2B/Fc. n = 10 / treatment group.

Table S1 – estimation of whole body fat volume at 12 weeks of age.

	**Number of mice**	**Mean**	**SEM**
**WT vehicle**	5	6.50	0.75
**WT ACVR2B/Fc**	5	5.18	0.38
**R6/2 vehicle**	5	7.02	0.87
**R6/2 ACVR2B/Fc**	5	7.54	0.20

Volume of each fat image that exceeded a threshold of five times the noise floor level. Statistical analysis was two-way ANOVA with post-hoc Bonferroni correction ($F_{(3,16)}$ = 2.76, p = 0.76).

Table S2

END-POINT ASSESSMENT SCALE	SCORE
**APPEARANCE**	
Normal	0
Emaciated	1
Emaciated AND (unkempt appearance OR facial piloerection OR squinting eyes)	2
**BODY WEIGHT**	
Normal (gaining weight)	0
<5% reduction	1
5 – 15% reduction	2
> 15% reduction	3
**DISEASE SCORE**	
Normal	0
Loss of fore-limb grip	1
Impaired paw placement on cage lid	2
**UNPROVOKED BEHAVIOUR**	
Normal	0
Minor depression	1
Disorientated behaviour OR relatively stationary	3

Score 1 – 3 normal
Score 4 – 7 monitor carefully
Score ≥ 8 – end stage

Adapted from 'A hand book of laboratory animal management and welfare'[2]

Table S3 - DESeq2 analysis of the RNAseq datasets

dataset	regulation	genes	GO	ENCODE TF ChIP-seq
Quad<br>WT veh vs.<br>R6/2 veh<br>(18558 genes<br>after filtering)	up	4052	chromatin modification<br>small GTPase mediated signal transduction<br>mitotic cell cycle	POLR2A, MYOD1,<br>MYOG, CTCF, UBTF
	down	3945	generation of precursor metabolites and energy<br>translation<br>gene expression	MYC, POLR2A, GATA1,<br>KAT2A, MYOG
Quad<br>WT veh vs. WT<br>ACVR2B/Fc<br>(18544 genes<br>after filtering)	up	241	mitotic cell cycle	FOXM1, ZNF384, E2F4,<br>POLR2A, EP300
	down	153	regulation of macrophage cytokine production<br>epithelial cell differentiation<br>extracellular matrix organization	n.s.
Quad<br>R6/2 veh vs.<br>R6/2<br>ACVR2B/Fc<br>(18557 genes<br>after filtering)	up	712	muscle filament sliding<br>branched-chain amino acid catabolic process<br>generation of precursor metabolites and energy	UBTF, MYOD1, POLR2A,<br>MYOG, CTCF
	down	538	extracellular matrix organization<br>ossification<br>positive regulation of smoothened signaling pathway	TCF12, MYOD1, MYOG,<br>TCF3, POLR2A
TA<br>WT veh vs.<br>R6/2 veh<br>(18544 genes<br>after filtering)	up	4006	chromatin modification<br>mitotic cell cycle<br>DNA repair	POLR2A, MYOG, CTCF,<br>UBTF, MYOD1
	down	3945	generation of precursor metabolites and energy<br>cellular amino acid metabolic process<br>translation	MYC, POLR2A, MYOG,<br>EP300, MAX
TA<br>WT veh vs. WT<br>ACVR2B/Fc<br>(18558 genes<br>after filtering)	up	609	mitotic cell cycle<br>muscle filament sliding<br>protein polyubiquitination	POLR2A, MYC, MYOD1,<br>GATA1, ZNF384
	down	546	extracellular matrix organization<br>axon guidance<br>ossification	EZH2, SUZ12, TCF12,<br>CBX2, CTCF
TA<br>R6/2 veh vs.<br>R6/2<br>ACVR2B/Fc<br>(18556 genes<br>after filtering)	up	363	transmembrane receptor protein serine/threonine kinase signaling<br>lipid transport<br>MAPK cascade	POLR2A, CTCF, UBTF,<br>ZC3H11A, EZH2
	down	259	muscle contraction	POLR2A, USF1, MYOD1,<br>TCF12, MYOG

Gene ontology (GO) and upstream regulator (ENCODE TF ChIP-seq) analysis of significantly up regulated (up) or down regulated (down) genes in the RNAseq datasets (dataset). The column ‘genes’ gives the number of dysregulated genes. Quad = quadriceps; TA = tibialis anterior. Veh = vehicle. n.s. = no significantly enriched terms.

Table S4 - DESeq2 analysis of ACVR2B/Fc treatment effects on genes that are dysregulated in R6/2 muscle

tissue	WT veh vs. R6/2 veh	R6/2 veh vs. R6/2 ACVR2B/Fc	genes	GO	ENCODE TF ChIP-seq
Quad	up	up	114	n.s.	RCOR1, EP300
		**down**	263	extracellular matrix organization<br>microtubule-based process	MYOD1, POLR2A, MAX, TEAD4, TCF12
	down	**up**	378	muscle filament sliding<br>mitochondrion<br>branched-chain amino acid catabolic process	MYOD1, MYOG, MAX, POLR2A, GATA1
		down	56	n.s.	TCF12, MYOG
TA	up	up	98	n.s.	POLR2A, STAT2, UBTF
		**down**	107	n.s.	USF1, POLR2A, MAX, HCFC1, USF2, MYOD1
	down	**up**	126	MAPK cascade<br>lipid transport	POLR2A
		down	61	muscle contraction<br>cellular amino acid metabolic process<br>cAMP catabolic process	n.s.

Gene ontology (GO) and upstream regulator (ENCODE TF ChIP-seq) analysis of the effects of ACVR2B/Fc treatment ‘column R6/2 veh vs. R6/2 ACVR2B/Fc’ on genes that are significantly up regulated (up) or down regulated (down) in the R6/2 muscle (no fold-change cut off), as indicated in column ‘WT veh vs. R6/2 veh’. The column ‘genes’ gives the number of dysregulated genes. Genesets highlighted in bold indicate genes that were corrected towards wild type levels by the ACVR2B/Fc treatment. Quad = quadriceps; TA = tibialis anterior. Veh = vehicle. n.s. = no significantly enriched terms.

Table S5 - WGCNA analysis of the RNAseq data

dataset	module	correlation (P_{adj})	genes	GO	ENCODE TF ChIP-seq
Quad<br>WT veh vs. WT ACVR2B/Fc (18544 genes after filtering))	navajowhite2	+ 0.75 (0.011)	653	hexose/glucose metabolism<br>tRNA aminoacylation<br>muscle filament sliding	MYOG, CTCF, MYOD1, BHLHE40, POLR2A
	mediumpurple3	- 0.75 (0.011)	156	phospholipid metabolic process<br>second-messenger-mediated signaling	n.s.
Quad<br>R6/2 veh vs. R6/2 ACVR2B/Fc (18557 genes after filtering))	antiquewhite4	+ 0.85 ($1.67 \cdot 10^{-5}$)	547	muscle filament sliding<br>hexose metabolic process<br>potassium ion transport	TCF12, MYOD1, CTCF, MAFK, MYOG
	orange	- 0.89 ($1.02 \cdot 10^{-4}$)	1069	extracellular matrix organization<br>positive regulation of cell development<br>synapse organization	TCF12, EZH2, MYOD1, RAD21, POLR2A
TA<br>WT veh vs. WT ACVR2B/Fc (18558 genes after filtering))	tan	+ 0.76 ($5.48 \cdot 10^{-3}$)	336	ribonucleoside diphosphate metabolic process<br>amine biosynthetic process<br>muscle filament sliding	MYOG, MYOD1, POLR2A, MAX, TCF12
	blue	- 0.67 (0.033)	1314	extracellular matrix organization<br>skeletal system development<br>regulation of vasculature development	EZH2, TCF12, CBX2, POLR2A, CBX8
TA<br>R6/2 veh vs. R6/2 ACVR2B/Fc (18556 genes after filtering)	lightcyan1	- 0.76 ($1.23 \cdot 10^{-3}$)	121	n.s.	MYC, MAX, KAT2A, YY1, HCFC1

Gene ontology (GO) and upstream regulator (ENCODE TF ChIP-seq) analysis of significantly (adjusted $p < 0.05$, Benjamini-Hochberg corrected) positively correlated (positive sign of correlation) or negatively correlated (negative sign of correlation) genes in the RNAseq datasets (dataset). The column module indicates the module name assigned by WGCNA. The column ‘genes’ gives the number of dysregulated genes. Quad = quadriceps; TA = tibialis anterior. Veh = vehicle. n.s. = no significantly enriched terms.

Table S6 - WGCNA analysis of ACVR2B/Fc treatment effects on genes that are dysregulated in R6/2 muscle

tissue	WT veh vs. R6/2 veh	R6/2 veh vs. R6/2 ACVR2B/Fc	genes	GO	ENCODE TF ChIP-seq
Quad	up	up	257	n.s.	EP300, CTCF, POLR2A, E2F1, UBTF
		**down**	700	extracellular matrix organization<br>regulation of neuron projection development<br>axon guidance	POLR2A, MYOG, MYOD1, UBTF, HCFC1
	down	**up**	723	generation of precursor metabolites and energy<br>branched-chain amino acid metabolic process<br>muscle filament sliding	MYOG, MAX, MYC, MYOD1, POLR2A
		down	144	endoplasmic reticulum unfolded protein response<br>monoamine transport	TCF12, MAX, MYOD1, MYOG, POLR2A
TA	up	up	312	mitotic nuclear division<br>response to type I interferon	FOXM1, UBTF, CTBP2, STAT2, POLR2A
		**down**	573	chromatin modification<br>mRNA splicing<br>DNA nucleotide-excision repair	POLR2A, KAT2A, HCFC1, GABPA, SIN3A
	down	**up**	454	branched-chain amino acid metabolic process<br>regulation of macrophage activation<br>regulation of humoral immune response mediated by circulating immunoglobulin	UBTF, MAFK, POLR2A, BHLHE40, CHD1
		down	282	muscle contraction<br>protein ADP-ribosylation	GATA1, POLR2A, EP300, MYOD1, MYC

Gene ontology (GO) and upstream regulator (ENCODE TF ChIP-seq) analysis of the effects of ACVR2B/Fc treatment ‘column R6/2 veh vs. R6/2 ACVR2B/Fc’ on genes that are significantly positively correlated (up) or negatively correlated (down) in the R6/2 muscle, as indicated in column ‘WT veh vs. R6/2 veh’. The column ‘genes’ gives the number of dysregulated genes. Genesets highlighted in bold indicate genes that were corrected towards wild type levels by the ACVR2B/Fc treatment. Quad = quadriceps; TA = tibialis anterior. Veh = vehicle. n.s. = no significantly enriched terms.

Table S7

Number of mice used per treatment group for each study and CAG repeat size (mean ± SD) for the R6/2 mice. Body weight and / or grip strength were always measured to ensure that there was comparable efficacy between batches of ACVR2B/Fc preparations.

		WT	R6/2	CAG	Body weight	Grip strength
Pilot experiment	vehicle	10	10	208 ± 3.1	√	√
	treated	10	10	208 ± 3.9		
Histology	vehicle	4	4	208 ± 2.1	√	√
	treated	4	4	209 ± 2.5		
MRI	vehicle	5	5	214 ± 6.0	√	√
	treated	5	4	209 ± 6.4		
Behaviour and end-stage assessment	vehicle	13	17	212 ± 4.3	√	√
	treated		14	217 ± 7.3		
Muscle physiology	vehicle	6	7	212 ± 5.5	√	n/a
	treated	4	6	212 ± 2.7		
Molecular analyses 12 weeks of age	vehicle	10	10	205 ± 5.3	√	√
	treated	10	10	205 ± 4.2		

√ indicates the assessments that were used to ensure that each batch of ACVR2/Fc showed comparable efficacy.

Table S8

Main effect tables from the two-way ANOVA analyses

	**Initial study – Figure 1 and Figure S1**									
	Body weight – males		Body weight - females		Grip strength - males		Grip strength - females		Body temp	
	F	*p*	F	*p*	F	*p*	F	*p*	F	*p*
Treatment group	$50.07_{(3,128)}$	***	$12.29_{(3,128)}$	***	$59.69_{(3,128)}$	***	$30.57_{(3,128)}$	***	$1.95_{(3,228)}$	0.121
Age	$27.61_{(7,128)}$	***	$21.78_{(7,128)}$	***	$55.29_{(7,128)}$	***	$65.82_{(7,128)}$	***	$5.54_{(7,228)}$	***
Treatment group x Age	$2.019_{(21,128)}$	0.009	$1.5_{(21,128)}$	0.088	$7.29_{(21,128)}$	***	$8.21_{(21,128)}$	***	$3.85_{(21,288)}$	***

	**Initial study – Figure 1 and Figure S1**									
	Tibialis weight		Gastrocnemius weight		Quadriceps weight		Brain weight		Heart weight / tibia length	
	F	*p*	F	*p*	F	*p*	F	*p*	F	*p*
Genotype	$38.96_{(1,36)}$	***	$39.95_{(1,36)}$	***	$59.89_{(1,36)}$	***	$61.74_{(1,36)}$	***	$14.13_{(1,36)}$	***
Treatment	$40.61_{(1,36)}$	***	$41.15_{(1,36)}$	***	$43.60_{(1,36)}$	***	$0.25_{(1,36)}$	0.621	$0.49_{(1,36)}$	0.486
Genotype x Treatment	$1.44_{(1,36)}$	0.238	$0.01_{(1,36)}$	0.917	$0.02_{(1,36)}$	0.888	$0.30_{(1,36)}$	0.591	$0.58_{(1,36)}$	0.45

	**Muscle Function – Figure 3**									
	EDL - time to peak		EDL – maximum twitch		EDL – half relaxation time		EDL – tetanic tension		EDL – number of motor units	
	F	*p*	F	*p*	F	*p*	F	*p*	F	*p*
Genotype	$19.22_{(2,41)}$	***	$0.26_{(2,41)}$	0.614	$27.93_{(2,41)}$	***	$0.31_{(2,41)}$	0.584	$1.70_{(2,41)}$	0.200
Treatment	$4.47_{(2,41)}$	0.041	$25.64_{(2,41)}$	***	$5.79_{(2,41)}$	0.021	$11.92_{(2,41)}$	***	$0.53_{(2,421)}$	0.470
Genotype x Treatment	$3.12_{(2,41)}$	0.085	$3.27_{(2,41)}$	0.078	$0.02_{(2,41)}$	0.889	$3.64_{(2,41)}$	0.063	$10.99_{(2,41}$	0.002

EDL = extensor digitalis longus

	**Muscle Function – Figure 3**							
	TA – time to peak		TA – maximum twitch		TA – half relaxation time		TA – tetanic tension	
	F	*p*	F	*p*	F	*p*	F	*p*
Genotype	$7.25_{(2,42)}$	0.01	$19.18_{(2,42)}$	***	$11.06_{(2,42)}$	0.002	$19.18_{(2,42)}$	***
Treatment	$0.003_{(2,42)}$	0.954	$22.36_{(2,42)}$	***	$5.67_{(2,42)}$	0.022	$22.36_{(2,42)}$	0.029
Genotype x Treatment	$0.23_{(2,42)}$	0.632	$3.53_{(2,42)}$	0.067	$3.68_{(2,42)}$	0.062	$3.53_{(2,42)}$	0.317

TA = tibialis anterior

	Behavioural study – Figure 4									
	Body weight		Fore-limb grip strength		Fore and hind limb grip strength		Rotarod performance		Activity	
	F	*p*	F	*p*	F	*p*	F	*p*	F	*p*
Treatment group	$115.20_{(2,492)}$	***	$145.63_{(2,490)}$	***	$220.98_{(2,490)}$	***	$21.13_{(2,204)}$	***	$77.16_{(2,234)}$	***
Age	$45.46_{(11,492)}$	***	$31.41_{(11,490)}$	***	$40.40_{(11,490)}$	***	$9.64_{(2,204)}$	***	$26.42_{(5,234)}$	***
Treatment group x Age	$4.25_{(22,492)}$	***	$9.83_{(22,490)}$	***	$14.41_{(22,490)}$	***	$4.76_{(8,204)}$	***	$1.32_{(10,234)}$	0.219

	Figure 5 and Figure S3					
	Analysis of DAPI nuclei		Analysis of total DAPI fluorescence		*Pax7* qPCR analysis	
	F	*p*	F	*p*	F	*p*
Genotype	$65.99_{(1,12)}$	***	56.82(1,12)	***	$103.79_{(1,25)}$	***
Treatment	$45.81_{(1,12)}$	***	30.73	***	$4.74_{(1,25)}$	0.038
Genotype x Treatment	$0.10_{(1,12)}$	0.753	0.1	0.757	$1.72_{(1,25)}$	0.2

***$p < 0.001$

Table S9

Multiple comparisons from the Bonferroni post-hoc correction of the ANOVA analyses

**Initial study – Figure 1 and Figure S1**								
**Age (weeks)**	**5**	**6**	**7**	**8**	**9**	**10**	**11**	**12**
**Body weight - males**								
WT-veh vs R6/2-veh	ns	ns	ns	ns	ns	ns	ns	**
WT-veh vs WT-ACVR2B/Fc	ns	ns	ns	ns	ns	ns	ns	*
R6/2-veh vs R6/2-ACVR2B/Fc	ns	ns	*	*	*	*	*	**
WT-veh vs R6/2-ACVR2B/Fc	ns	ns	ns	ns	ns	ns	ns	ns
**Body weight - females**								
WT-veh vs R6/2-veh	ns	ns	ns	ns	ns	ns	ns	ns
WT-veh vs WT-ACVR2B/Fc	ns	ns	ns	ns	ns	ns	ns	ns
R6/2-veh vs R6/2-ACVR2B/Fc	ns	ns	ns	ns	ns	ns	ns	*
WT-veh vs R6/2-ACVR2B/Fc	ns	ns	ns	ns	ns	ns	ns	ns
**Fore limb grip strength - males**								
WT-veh vs R6/2-veh	ns	ns	ns	ns	ns	ns	***	***
WT-veh vs WT-ACVR2B/Fc	ns	ns	ns	ns	ns	ns	**	***
R6/2-veh vs R6/2-ACVR2B/Fc	ns	ns	ns	ns	**	*	***	***
WT-veh vs R6/2-ACVR2B/Fc	ns	ns	ns	ns	ns	ns	ns	ns
**Fore limb grip strength - females**								
WT-veh vs R6/2-veh	ns	ns	ns	ns	ns	ns	**	***
WT-veh vs WT-ACVR2B/Fc	*	ns	ns	ns	*	*	**	**
R6/2-veh vs R6/2-ACVR2B/Fc	ns	ns	ns	ns	**	**	***	***
WT-veh vs R6/2-ACVR2B/Fc	ns	ns	ns	ns	**	ns	ns	ns
**Body temperature – males and females**								
WT-veh vs R6/2-veh	ns	ns	ns	ns	ns	ns	ns	ns
WT-veh vs WT-ACVR2B/Fc	*	**	ns	ns	ns	ns	ns	ns
R6/2-veh vs R6/2-ACVR2B/Fc	ns	ns	ns	ns	ns	ns	ns	ns
WT-veh vs R6/2-ACVR2B/Fc	ns	*	*	ns	ns	ns	ns	ns

**Tissue Weights**					
	Quad	Gast	Tibialis	Brain	Heart weight/ tibia length
WT-veh vs R6/2-veh	***	***	**	***	ns
WT-veh vs WT-ACVR2B/Fc	***	***	***	ns	ns
R6/2-veh vs R6/2-ACVR2B/Fc	***	***	**	ns	ns
WT-veh vs R6/2-ACVR2B/Fc	ns	ns	ns	***	ns

*$p < 0.05$; **$p < 0.01$; ***$p < 0.001$

**Muscle function – Figure 3**					
EDL	time to peak	max twitch	half relax time	tetanic tension	motor unit number
WT-veh vs R6/2-veh	***	ns	***	ns	**
WT-veh vs WT-ACVR2B/Fc	ns	ns	ns	ns	ns
R6/2-veh vs R6/2-ACVR2B/Fc	*	***	ns	***	*
WT-veh vs R6/2-ACVR2B/Fc	ns	**	ns	ns	ns
TA	time to peak	max twitch	half relax time	tetanic tension	
WT-veh vs R6/2-veh	ns	***	***	***	
WT-veh vs WT-ACVR2B/Fc	ns	ns	ns	ns	
R6/2-veh vs R6/2-ACVR2B/Fc	ns	***	**	ns	
WT-veh vs R6/2-ACVR2B/Fc	ns	ns	ns	ns	

TA = tibialis anterior
EDL = extensor digitalis longus
ns = not significant

**Behavioural study – Figure 4**												
**Age (weeks)**	**4**	**5**	**6**	**7**	**8**	**9**	**10**	**11**	**12**	**13**	**14**	**15**
**Body weight**												
WT-veh vs R6/2-veh	ns	ns	ns	ns	ns	ns	ns	*	ns	ns	ns	***
R6/2-veh vs R6/2-ACVR2B/Fc	ns	ns	ns	ns	ns	ns	ns	ns	**	*	ns	ns
WT-veh vs R6/2-ACVR2B/Fc	ns	ns	ns	ns	ns	ns	ns	***	**	*	ns	ns
**Fore limb grip strength**												
WT-veh vs R6/2-veh	ns	ns	ns	ns	ns	ns	ns	ns	***	***	***	***
R6/2-veh vs R6/2-ACVR2B/Fc	ns	ns	ns	ns	***	***	***	***	***	***	***	***
WT-veh vs R6/2-ACVR2B/Fc	ns	ns	*	ns	**	***	**	**	ns	*	ns	ns
**Fore and Hind-limb grip strength**												
WT-veh vs R6/2-veh	ns	ns	ns	ns	ns	ns	*	*	***	***	***	***
R6/2-veh vs R6/2-ACVR2B/Fc	ns	ns	*	**	**	**	***	***	***	***	***	***
WT-veh vs R6/2-ACVR2B/Fc	ns	ns	*	**	ns	ns	*	**	***	**	ns	ns
**Rotarod performance**												
WT-veh vs R6/2-veh	ns		ns		*		***		***			
R6/2-veh vs R6/2-ACVR2B/Fc	ns		ns		ns		ns		ns			
WT-veh vs R6/2-ACVR2B/Fc	ns		ns		**		*		***			
**Locomotor activity**												
WT-veh vs R6/2-veh		ns		*		*		***		***		***
R6/2-veh vs R6/2-ACVR2B/Fc		ns		ns		ns		ns		ns		ns
WT-veh vs R6/2-ACVR2B/Fc		ns		**		***		***		***		***

*$p < 0.05$; **$p < 0.01$; ***$p < 0.001$

REFERENCES

1 Bernstein, M. A., King, K. F. & F., Z. X. *Handbook of MRI pulse sequences*. (Elsevier Academic Press, 2004).

2 Wolfensohn, S. & Lloyd, M. *A handbook of laboratory animal management and welfare*. 3rd edn, 62 (Blackwall Publishing, 2003).
